# Multimodal spatial omics: From data acquisition to computational integration

**DOI:** 10.1016/j.patter.2026.101592

**Published:** 2026-06-30

**Authors:** Esra Busra Isik, Yusuf Hakan Usta, Maryam Riazi, Haozhe Liu, William Roach, Hongpeng Zhou, Anna Nicolaou, Magnus Rattray, Sokratia Georgaka

**Affiliations:** 1 Division of Informatics, Imaging & Data Sciences, School of Health Sciences, University of Manchester, Manchester, UK; 2 Department of Computer Science, University of Manchester, Manchester, UK; 3 Department of Microbiology, University Hospitals Tees, Middlesbrough, UK; 4 Division of Pharmacy & Optometry, School of Health Sciences, and Lydia Becker Institute of Immunology and Inflammation, Faculty of Biology, Medicine and Health, University of Manchester, Manchester, UK

**Keywords:** spatial omics, multimodal integration, computational methods, deep learning, matrix factorization, optimal transport

## Abstract

Recent developments in spatial omics technologies have enabled the generation of high-dimensional molecular data, including transcriptomics, proteomics, and epigenomics, within their spatial tissue context, either through co-profiling on the same slice or through profiling across serial tissue sections. These datasets, which are often complemented by images, have given rise to multimodal frameworks that capture both the cellular and architectural complexity of tissues across multiple molecular layers. Integration of such multimodal data poses significant computational challenges due to differences in scale, resolution, and data modality. In this review, we present a comprehensive overview of computational methods developed to integrate multimodal spatial omics and imaging datasets. We highlight key algorithmic principles underlying these methods, ranging from probabilistic to the latest deep learning approaches.

## The bigger picture

Understanding how cells function within tissues requires more than knowing which molecules they contain; it also depends on where those molecules are located and how cells interact with their neighbors. Spatial omics technologies have transformed biology by enabling researchers to measure gene expression, proteins, metabolites, and other molecular layers directly within intact tissues by preserving their spatial organization. This is particularly important in diseases such as cancer, where the spatial architecture of cells within a tumor can influence how the disease progresses and responds to treatment. No single technology, however, can capture the full complexity of a biological system. Different methods measure different aspects of cellular function, such as genes, proteins, or regulatory elements, often at different resolutions and from separate tissue sections. Integrating these diverse datasets into a unified view presents a major computational challenge, requiring new methods that can reconcile differences in scale, noise, and data structure. The ability to integrate spatial multi-omics data has the potential to transform biomedical research and clinical practice. It could enable more precise disease diagnosis, reveal new therapeutic targets, and support the development of predictive models that simulate how tissues respond to treatment. As technologies and computational methods continue to advance, spatial multi-omics is poised to become a central tool for understanding biology in its native context.

In this review, we bring together both experimental and computational approaches to multimodal spatial omics. We explain how researchers can combine information across molecular layers to better understand tissue organization, and we outline the key algorithmic frameworks, ranging from statistical models to deep learning, that make this integration possible. By clarifying these concepts and highlighting current limitations and future directions, we aim to make this rapidly evolving field more accessible to a broad scientific audience.

## Teaser

Multimodal spatial omics technologies enable mapping of molecular features within tissues, but integrating these diverse data remains challenging. This review outlines experimental strategies and computational frameworks, highlighting how combining modalities can reveal tissue organization and advance biological discovery, while also outlining key challenges and future directions.

## Introduction

Spatial omics technologies have fundamentally changed our ability to study biology by enabling molecular profiling of transcriptomes, proteomes, epigenomes, and metabolomes while preserving the spatial organization of cells within their native microenvironment.^1^^,^^2^ Unlike conventional bulk or single-cell approaches that require tissue dissociation, spatial methods retain the architectural context vital for understanding how cellular phenotypes emerge from local interactions, signaling gradients, and microenvironmental niches.^3^ This spatial dimension has proven critical across biomedicine: in oncology, it reveals the heterogeneous organization of tumor microenvironments that influences therapeutic response; in developmental biology, it maps the molecular gradients that pattern tissue formation; in neuroscience, it charts the regional specialization of brain circuits.^4^ However, no single spatial modality captures the full complexity of a cellular state. Transcriptomics captures active gene programs but does not fully capture post-transcriptional regulation, proteomics quantifies functional effectors but misses the regulatory events that produced them, epigenomics exposes chromatin landscapes but not their downstream consequences, and metabolomics reflects cellular biochemistry but requires pathway models for interpretation.^2^^,^^5^ Increasingly, researchers recognize that biological insight demands integration across these molecular layers—linking chromatin accessibility to transcription, protein abundance, and metabolic output within the same spatial context. This recognition has driven the rapid development of experimental technologies for multimodal spatial profiling, as well as computational frameworks for cross-modal integration.

Integrating multimodal spatial data poses significant computational challenges that distinguish it from conventional unimodal omics analysis. Spatial omics modalities differ fundamentally in resolution, feature dimension, and data structure. In addition, when modalities are acquired from serial tissue sections rather than co-profiled on the same slice, spatial correspondence must be computationally inferred through co-registration algorithms that introduce additional uncertainty.^6^

**Figure 1:**
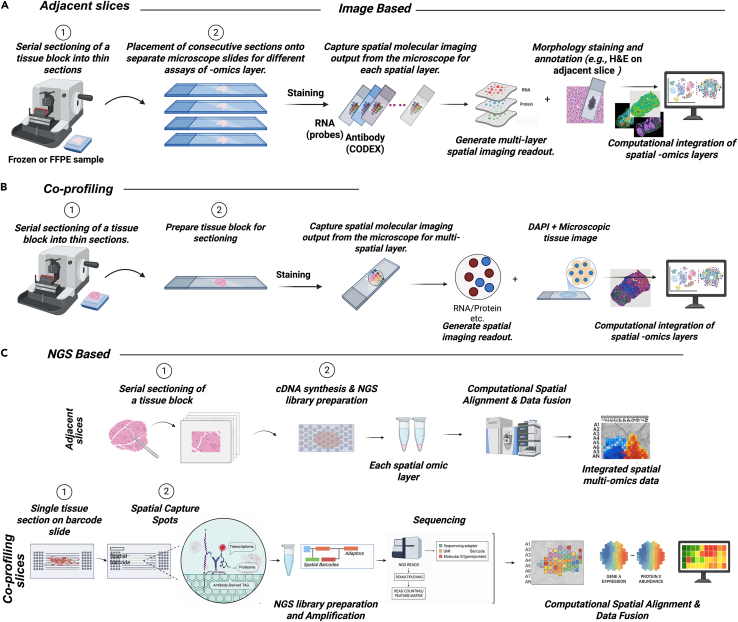
Experimental workflows for spatial multimodal data acquisition (A) Serial sectioning: consecutive tissue sections are profiled with different spatial omics technologies and computationally co-registered to a common coordinate system. (B) Co-profiling: multiple molecular layers are measured simultaneously on the same tissue section, preserving exact spatial correspondence without computational alignment. (C) Sequencing-based (left) versus imaging-based (right) spatial omics workflows.

In this Review, we provide a comprehensive overview of multimodal spatial omics integration—from experimental data generation to computational analysis to outstanding challenges. We begin by surveying experimental acquisition strategies, contrasting two fundamental approaches: co-profiling methods that measure multiple modalities from the same tissue section and serial-section workflows that maximize per-modality quality at the cost of requiring computational alignment (Figure 1). We cover sequencing-based and imaging-based platforms across spatial omics modalities, highlighting their respective strengths and limitations. We then introduce key concepts for understanding multimodal data integration, including integration strategies and fusion approaches, before systematically reviewing computational frameworks organized by algorithmic family: probabilistic and statistical inference, matrix factorization and latent variable models, optimal transport (OT) and geometric alignment, and deep learning approaches. Throughout, we contextualize methods according to the key computational tasks they address (Box 1): deconvolution of cell-type mixtures, identification of spatial domains, co-registration of serial sections, prediction of molecular profiles from histology images, and gene imputation. We identify fundamental challenges that must be overcome for the field to mature, including standardization of protocols and data formats, development of rigorous benchmarks with ground-truth, scalability to terabyte-scale datasets, and interpretable models that connect computational abstractions to testable biological hypotheses. We conclude by highlighting priorities for future development.

Box 1 **Key computational tasks**

**Co-registration of modalities**

Spatial omics datasets obtained from serial tissue sections must be co-registered to a common coordinate system before integration, with histology-stained images often serving as proxies for this co-registration.

**Deconvolution**

Most sequencing-based ST technologies capture mRNA at multicellular rather than single-cell resolution (for example, 10× Visium, Slide-seq). As a result, each spot can contain a mixture of cell types. Computational deconvolution methods have therefore been developed that infer the cell-type composition at each spatial location. Most of these approaches can be viewed as multimodal: some rely on annotated single-cell RNA sequencing (scRNA-seq) reference datasets that link cell-type-specific transcriptomic profiles to spatial spots, while others integrate histological image features extracted from paired H&E-stained images to improve deconvolution performance.

**Spatial domain identification**

In spatial omics, spatial domains are contiguous tissue regions where cells share similar molecular expression patterns and biological functions. These domains are biologically important as they can capture distinct cell states, disease-associated regions, or microenvironments, as well as distinct functional areas such as fibrotic or hypoxic niches. Integrating multiple spatial omics modalities to identify spatial domains provides a deeper insight into tissue organization and function.

**Prediction of omics from histology images**

Histology imaging is routinely performed in clinical practice and serves as a cornerstone of medical diagnosis. In contrast, spatial omics assays remain costly and are rarely implemented in clinical settings. With the growing availability of paired histology and spatial omics datasets, deep learning methods have emerged that can predict spatially resolved transcriptomic and proteomic profiles directly from H&E-stained images. Most of these approaches adopt a tile-based representation of the H&E image, whereby whole-slide images are segmented into fixed-size patches that are spatially aligned to the resolution of the underlying spatial omics technology, most commonly matching the diameter of a 10× Visium spot. These image patches are then used as inputs to deep learning models to learn mappings between morphological and molecular features. These approaches aim to leverage histology as a low-cost proxy for expensive spatial omics measurements.

**Gene imputation and cross-modal translation**

*In situ* hybridization-based ST techniques provide single-cell-resolved spatial gene expression but they are limited by low gene throughput. Gene imputation methods address this limitation by leveraging scRNA-seq reference datasets combined with ST to infer spatial gene expression of genes that are not directly measured.

Cross-modal translation methods typically rely on generative models trained on paired spatial multimodal datasets to learn correspondences between spatial modalities and predict one modality from another. These approaches enable multimodal spatial omics integration in settings where one or more modalities are missing.

## Overview of spatial multimodal acquisition strategies

Spatial multimodal omics acquisition aims to capture multiple molecular and phenotypic layers from tissue while preserving their spatial organization. Broadly, current strategies fall into two categories: (1) measurements performed on adjacent tissue sections and (2) simultaneous measurements on the same tissue section, through co-profiling. These strategies differ in experimental design, technical complexity, and in the degree to which the spatial correspondence between modalities must be reconstructed computationally.

In serial sectioning workflows, consecutive tissue sections are processed using different spatial omics technologies, and the resulting datasets are computationally co-registered to a common coordinate system (Figure 1A). This approach is widely used because it allows each modality to be generated using its optimal protocol. Within this framework, spatial omics platforms can be broadly divided into sequencing-based and imaging-based technologies, each with distinct experimental workflows and data characteristics (Figure 1C).

By contrast, co-profiling strategies perform simultaneous measurements of multiple molecular modalities in the same tissue section, eliminating the need for computational co-registration and preserving exact spatial correspondence between modalities (Figure 1B). Similar to serial sectioning approaches, co-profiling technologies can also be categorized into sequencing-based and imaging-based platforms, reflecting differences in molecular capture, readout mechanisms, and data structure.

### Measurements on adjacent tissue slices

We begin by discussing sequencing-based spatial profiling technologies, followed by imaging-based approaches.

#### Sequencing-based ST

Sequencing-based spatial transcriptomics (ST) methods typically rely on array-based capture slides coupled with next-generation sequencing (NGS) to localize mRNA molecules within the spatial context of the tissue architecture. Spatially resolved transcriptomics was first introduced with ST technology, which uses a glass slide coated with an array of 1,007 spatially barcoded capture probes (“spots”) with a diameter of 100 μm containing oligo(*d*)T primers to bind mRNA molecules from the tissue sections.^7^ This approach was later refined by the 10× Genomics Visium platform, which increased the resolution to 5,000 spots (55 μm diameter). Recently, the 10× Visium HD platform further advanced ST to near single-cell resolution by introducing slides with increased oligonucleotide barcode density.^8^ These technologies are accompanied by high-resolution hematoxylin and eosin (H&E)—stained images acquired from the same tissue section, enabling direct integration of morphological and transcriptomic features.

Another group of sequencing-based technologies builds on similar core principles but employs DNA-barcoded microparticles (“beads”) to capture spatially resolved gene expression profiles. Slide sequencing (Slide-seq) introduced a strategy where 10 μm DNA-barcoded beads are randomly deposited on a rubber-coated glass slide to form a dense array termed a “puck.”^9^ While Slide-seq offers a highly resolved ST method, its low transcript detection sensitivity limits application across a wide range of biological problems. Slide-seq V2 addresses this limitation by offering an order of magnitude higher sensitivity through enhancements in the barcoded bead synthesis, array indexing and library preparation, enabling broader application of this technology.^10^ High-definition ST (HDST) relies on a similar strategy, using a randomly ordered bead array in which barcoded poly(*d*)T oligonucleotides are deposited onto 2 μm wells.^11^ Unlike Slide-seq technologies, HDST provides matched H&E images. More recently, Curio Bioscience has commercialized and extended the Slide-seq concept through two complementary platforms.^12^ Curio Seeker uses 10 μm DNA-barcoded beads arranged in a tightly packed monolayer on tiles of either 3 × 3 mm or 10 × 10 mm, enabling whole-transcriptome spatial profiling at single-cell resolution. Curio Trekker takes a distinct approach: tissue sections are placed on spatially barcoded tiles, and UV-cleavable oligonucleotides are released and attached to individual nuclei, which are then processed through standard single-nucleus RNA sequencing (RNA-seq) workflows. By assigning spatial barcodes directly to nuclei, Trekker bypasses the need for computational deconvolution, offering single-nucleus spatial resolution in approximately 1 h of upstream processing time.

#### Sequencing-based spatial epigenomics

The spatial organization of the epigenome—comprising DNA regulatory elements and chromatin modifications—is crucial for understanding gene regulation in development and disease. Only recently have methods emerged to profile epigenetic marks with spatial resolution.^13^ Pioneering techniques are adapting assays such as ATAC-seq (for chromatin accessibility) and cleavage under targets and tagmentation (CUT&Tag) (for histone modifications or transcription factor binding) to tissue sections.^14^

In the standard ATAC-seq approach, transposase enzymes preferentially access open chromatin regions and insert sequencing adapters to profile chromatin accessibility. Spatial assay for transposase-accessible chromatin using sequencing (ATAC-seq) extends this approach to fixed tissue sections by introducing transposases *in situ*, followed by spatial barcoding (often with a DBiT-seq style microfluidic grid) to index the DNA fragments by location. In this approach, transposase (Tn5) flows across the tissue to fragment DNA *in situ*, then two sets of barcodes (A and B channels) are applied to the tissue in a manner similar to DBiT-seq. Sequencing these fragments yields a map of open chromatin regions across the tissue. Spatial ATAC-seq has achieved ∼20 μm resolution (roughly single-cell level) in tissue sections.

(CUT&Tag) is an assay in which an antibody targeting a specific histone modification directs a transposase to genomic sites of interest, where DNA is cleaved and tagged for sequencing. Spatial CUT&Tag extends this by performing antibody binding directly in tissue sections, followed by microfluidic barcoding to assign spatial coordinates. The result is a spatial map of histone mark enrichment across the tissue. A breakthrough study applied spatial CUT&Tag to mouse embryos for three key marks (H3K4me3 active promoters, H3K27ac active enhancers, and H3K27me3 repressive heterochromatin) at ∼20 μm resolution. It revealed that developing brain regions exhibit distinct epigenetic landscapes: certain neuronal layers show strong H3K27ac enrichment at specific enhancers associated with neuronal subtype specification, whereas other regions showed H3K27me3-mediated repression in terminally differentiated cells. In another application, spatial CUT&Tag applied to diseased tissue could highlight fibrotic regions with abnormal chromatin states, helping to explain pathological gene expression patterns. Nevertheless, a key challenge of these methods is data sparsity. Even single-cell ATAC-seq exhibits considerable sparsity, which can be further exacerbated in tissue contexts. Aggregating data across neighboring cells within a local region can help mitigate this limitation.^15^^,^^16^^,^^17^

Together, these sequencing-based approaches provide powerful genome-wide spatial readouts but are often limited by resolution or sensitivity, motivating complementary imaging-based strategies.

#### Imaging-based ST

*In situ* hybridization (ISH) techniques enable the quantification, localization, and visualization of individual RNA molecules through nucleic acid probes complementary to target RNA sequences.^18^ Early ISH methods employed probes labeled with radioactive isotopes, such as tritium. These were later replaced by fluorescently labeled probes, leading to improved resolution, enhanced safety and easier imaging. This advancement led to fluorescence *in situ* hybridization (FISH) and subsequently to single-molecule FISH (smFISH), which enables the detection and quantification of individual RNA transcripts within the cells. Because each gene requires a unique fluorophore, this approach is limited to a small number of genes (typically 3–5). Sequential FISH (seqFISH) and its improved version, seqFISH+, are extensions of smFISH that enable the detection of thousands of genes through iterative reuse of a limited set of fluorophores across multiple rounds of hybridization.^19^^,^^20^

An alternative method that overcomes the limitations of the smFISH approach is multiplexed error-robust FISH (MERFISH). Instead of assigning each gene a unique fluorophore or reusing the fluorophores across multiple cycles, MERFISH uses combinatorial labeling and sequential imaging, where error-robust binary barcodes are assigned to individual RNA species and decoded through sequential hybridization rounds.^21^ Key commercially available FISH-based platforms include Vizgen’s Merscope (MERFISH), which provides 100 nm spatial resolution and profiles up to 1,000 genes, and NanoString CosMx (smFISH), which enables whole-transcriptome mapping (∼19,000 genes) at cellular or subcellular resolution.

*In situ* sequencing (ISS) is another imaging-based technology that enables the direct sequencing of RNA molecules within tissue. ISS relies on barcoded padlock probes that hybridize to specific RNA targets, followed by ligation and amplification through rolling circle amplification, generating rolling circle products that can be visualized by imaging-based sequencing methods.^22^ Xenium, a commercially available ISS-based platform provided by 10× Genomics, provides subcellular resolution for panels of up to 5,000 genes.^23^

Overall, imaging-based techniques provide single-cell or subcellular spatial resolution but typically rely on predefined, targeted gene panels. In contrast, sequencing-based platforms enable genome-wide gene expression profiling, making them well suited for exploratory analysis but generally at the cost of lower spatial resolution. Nevertheless, continued advances in spatial technologies are improving both gene coverage and spatial resolution, thereby reducing the trade-offs between these approaches. An additional advantage of sequencing-based methods is their ability to capture splice isoforms, making them well suited for applying RNA velocity algorithms. Conversely, imaging-based platforms typically exhibit higher capture efficiency at greater sensitivity and specificity than sequencing-based techniques.

#### Imaging-based spatial epigenomics

In spatial epigenomics, chromatin tracing uses sequential DNA FISH to trace the three-dimensional paths of chromosomes in nuclei, providing a spatial map of genome folding (a technique sometimes called “Hi-M” for *in situ* Hi-C visualization). These have shown how certain loci come together in “hubs” inside the nucleus of specific cell types. This imaging technique achieves very high-resolution (∼30 nm between probes) and has been used to map the 3D genome at single-cell resolution in tissues, although its throughput remains limited.^24^

#### Imaging-based spatial proteomics

Imaging-based spatial proteomics encompasses a diverse set of technologies to visualize and quantify proteins directly within intact tissues while preserving spatial context. These approaches differ in the way the proteins are identified, the imaging modality used, and their achievable spatial resolution and multiplexing capacity. Broadly, spatial proteomics technologies can be divided into label-free approaches based on mass spectrometry imaging (MSI) and approaches that use antibodies, metal tags, or DNA barcodes to identify and localize cell-specific protein markers. Label-free MSI platforms such as MALDI-MSI can be coupled to QTOF (quadrupole time-of-flight) analyzers or combined with laser microdissection and liquid chromatography-tandem mass spectrometry (LC-MS/MS) to analyze the proteins and peptides of interest. Antibody-based technologies include cyclic fluorescence imaging, DNA-barcoded antibody imaging, metal-tagged immunohistochemistry combined with MSI, and region-based digital profiling strategies.

Multiplexed fluorescence imaging remains a widely used strategy and relies on repeated cycles of antibody staining and imaging to overcome the limited spectral range of fluorophores. In each cycle, fluorophore-conjugated antibodies are applied, imaged, and subsequently quenched or removed prior to the introduction of the next set of markers. Techniques such as array tomography,^25^ MELC,^26^ MxIF,^27^ CycIF,^28^ t-CyCIF,^29^^,^^30^ 4i,^31^ and IBEX^32^^,^^33^ have been widely applied across different tissue types and are particularly valuable for mapping the tumor immune microenvironment. These approaches typically achieve subcellular resolution, but high-plex experiments require many staining cycles, increasing experimental duration and potentially leading to antigen loss, incomplete signal quenching, and antibody cross-reactivity. The large dynamic range of protein abundance further challenges fluorescence-based detection sensitivity.^34^ Recent developments have introduced automated implementations that improve throughput and reproducibility. For example, microfluidic-based systems such as Lunaphore COMET enable sequential immunofluorescence staining through controlled delivery and removal of antibodies within a microfluidic chamber, allowing multiplex detection of dozens of proteins while maintaining tissue morphology.^35^

DNA-barcoded antibody imaging approaches improve scalability by separating antigen recognition from signal detection. In these methods, tissues are stained once using antibodies conjugated to unique DNA barcodes, after which complementary fluorescent oligonucleotides are sequentially hybridized and removed to visualize individual markers. Techniques such as Exchange-PAINT,^36^ DEI,^37^ and CODEX^38^^,^^39^ exemplify this strategy. In CODEX (co-detection by indexing), iterative hybridization of fluorescent probes allows simultaneous imaging of tens to over one hundred protein markers within intact tissues. This technology has been commercialized as the PhenoCycler platform (Akoya Biosciences) and has been widely used to characterize cellular interactions within the tumor microenvironment. Signal amplification strategies can further increase sensitivity for low-abundance proteins. For example, Immuno-SABER^40^ and isHCR^41^ amplify signals from DNA-tagged antibodies through programmable DNA concatemers, while hybrid approaches such as SABER-imaging mass cytometry (IMC) combine DNA barcoding with IMC to expand multiplexing capacity.^42^

Mass spectrometry-based imaging technologies provide an alternative strategy for highly multiplexed spatial proteomics. By attaching ionizable metal isotopes to antibodies, these approaches enable “next-generation immunohistochemistry” that is detected using MSI rather than fluorescence.^43^ In multiplexed ion beam imaging (MIBI), a primary ion beam releases secondary ions from metal-labeled antibodies for magnetic sector or TOF analysis, enabling the detection of ∼40 protein markers while revealing the tumor-immune architecture at subcellular resolution.^44^^,^^45^^,^^46^ High-definition MIBI further improves spatial resolution to approximately 260–1,000 nm.^46^^,^^47^ The IMC platform instead uses laser ablation to vaporize tissue regions and deliver metal tags to a TOF mass cytometer, analogous to CyTOF single-cell assays.^48^ IMC enables simultaneous detection of dozens of proteins and post-translational modifications within tissue sections,^49^ and can be combined with RNA detection approaches such as RNAscope to investigate mRNA-protein concordance in tumor tissues.^50^ Although these methods provide high multiplexing with minimal spectral overlap, they typically require vacuum-compatible samples and involve destructive acquisition.

Another class of technologies profiles spatial protein expression within defined tissue regions rather than generating pixel-level images. The GeoMx digital spatial profiler (DSP) uses antibodies conjugated to photocleavable oligonucleotide barcodes that are released upon targeted ultraviolet illumination of selected regions of interest.^51^ The released oligonucleotide tags are then quantified using digital counting or NGS, enabling multiplex measurement of protein panels in formalin-fixed paraffin-embedded (FFPE) tissue while preserving spatial information at the region level.

Label-free LC-MS/MS ST combines sensitive MS with precise tissue sampling, frequently through laser capture microdissection (LCM).^52^^,^^53^^,^^54^^,^^55^^,^^56^^,^^57^ Pixel-by-pixel strategies divide tissues into small voxels that are processed individually, allowing quantification of hundreds to thousands of proteins per voxel.^58^^,^^59^^,^^60^^,^^61^ Region of interest approaches instead focus on histologically defined areas, as demonstrated by LCM-SISPROT, which profiles specific cell populations from very small tissue regions.^62^^,^^63^ Deep visual proteomics integrates high-resolution microscopy, AI-based cell classification, and ultrasensitive MS to map thousands of proteins at near single-cell resolution.^64^ These workflows enable the generation of large-scale spatial protein atlases across tissues and disease states.^65^

MSI also provides untargeted and label-free strategies for mapping protein distributions across tissues. MALDI is the most widely used MSI technique for spatial proteomics, as it can ionize proteins directly from tissue surfaces. In these approaches, mass spectra are acquired directly from defined spatial coordinates, allowing reconstruction of protein abundance maps.^66^^,^^67^ Top-down MSI analyses intact proteins using techniques, such as MALDI, nanoDESI (desorption electrospray ionization), or LESA,^68^^,^^69^^,^^70^ and recent technological advances have enabled near-cellular spatial resolution in human tissues.^71^^,^^72^^,^^73^ Bottom-up MSI workflows digest proteins *in situ* and image the resulting peptides,^74^ which is particularly useful for FFPE samples where the formalin-induced crosslinking must first be reversed and proteins digested with enzymes such as trypsin.^75^ Workflow optimizations and enzyme combinations further improve coverage,^76^ while LC-MS/MS analysis of extracted and digested proteins from adjacent sections can assist with peptide identification.^77^^,^^78^^,^^79^

Taken together, these spatial proteomics technologies offer complementary strengths and limitations in terms of spatial resolution, multiplexing capacity, and proteome coverage. Imaging-based antibody approaches typically achieve single-cell or subcellular resolution but are constrained by the number of detectable markers, whereas MSI-based workflows enable broader proteome coverage at the cost of reduced spatial resolution or destructive acquisition. Integrating these complementary technologies is therefore increasingly important for obtaining comprehensive spatial maps of protein expression across complex tissues.

Notably, spatial proteomics panels can also provide an indirect readout of metabolic activity by including antibodies against key metabolic enzymes and transporters. For example, antibody panels targeting glycolytic enzymes (e.g., HK2, PKM2, and LDHA), oxidative phosphorylation complexes, or transporters (e.g., SLC1A5 and SLC7A5) enable spatially resolved inference of metabolic states at single-cell resolution.^80^ These enzyme-level readouts complement direct metabolite mapping by MSI, which captures metabolic products but lacks the cellular resolution and molecular specificity required for individual enzymatic steps. The combination of spatial proteomics panels with spatial metabolomics thus offers a multi-layered view of tissue metabolism, linking enzyme expression to metabolic output within the same spatial context.

#### Imaging-based spatial metabolomics

Metabolites and small molecules present another frontier for spatial omics. While not explicitly mentioned alongside transcriptomics and proteomics as frequently, spatial metabolomics refers to mapping the distribution of metabolites (like lipids, sugars, signaling molecules, and drug compounds) within tissues. The primary technologies for this are in the realm of MSI, as methods analogous to FISH do not exist for small molecules (they are too varied and generally cannot be tagged *in situ* without perturbation). MALDI MSI, already described above, can detect a broad array of metabolites in tissue without labels,^81^ although the detection of small metabolites can be hindered by matrix interference, which represents a major disadvantage for metabolomics applications. DESI imaging, a softer ionization technique, uses a stream of charged solvent droplets to desorb molecules from the tissue surface for MS analysis. DESI is particularly well suited for profiling lipids and small molecules directly from tissue at ∼50–200 μm resolution and has the advantage of minimal sample preparation (no matrix coating needed).^82^^,^^83^ Although no single imaging method captures all metabolites, MS-based approaches together provide a powerful toolkit for spatial metabolite mapping.^13^ The associated data analysis often involves overlaying metabolite maps with anatomical features to interpret, for example, how a drug is distributed in a tumor or which metabolic pathways are active in a microenvironment. For computational biologists, spatial metabolomics raises interesting challenges in spectral deconvolution and metabolite identification directly from tissues, as well as integration with transcriptomic or proteomic maps (to link enzymes with their products spatially). Although still a specialized field, spatial metabolomics is becoming important in pharmacology (drug localization and effect mapping) and pathology (metabolic reprogramming in disease contexts).

### Simultaneous measurements on the same tissue section

#### ST and spatial proteomics

Recent advances in spatial omics technologies have enabled the simultaneous detection of transcripts and proteins within the same tissue section. These multimodal approaches extend ST platforms by incorporating antibody-based protein detection, enabling joint profiling of gene expression and protein abundance. Such technologies generally fall into two categories: sequencing-based approaches that integrate antibody-derived tags with spatial barcoding strategies, and imaging-based platforms that combine multiplex RNA detection with antibody-based protein imaging.

Sequencing-based multimodal technologies typically extend ST workflows by incorporating oligonucleotide-conjugated antibodies that generate antibody-derived tags (ADTs). The standard 10× Genomics Visium platform supports whole-transcriptome profiling together with immunofluorescence detection of a small number of protein markers. The Visium CytAssist workflow expands this approach by enabling spatially barcoded capture of mRNA alongside oligo-conjugated antibodies for protein profiling. Although transcriptome-wide RNA detection is supported, protein measurements are typically limited to a validated antibody panel of approximately 35 markers with the possibility of adding custom antibodies that require careful optimization. Spatial protein and transcriptome sequencing (SPOTS) combines Visium’s poly(A)-based capture chemistry with DNA-barcoded antibodies to simultaneously measure whole-transcriptome RNA expression together with panels of more than 30 proteins.^84^ Similarly, spatial multi-omics (SM-omics) builds on the original ST technology to enable transcriptome-wide RNA profiling while integrating fluorescence or DNA-barcoded antibodies for the detection of six proteins, although the spatial resolution remains relatively coarse (∼100 μm).^85^ Spatial-CITE-seq further extends this strategy by staining tissue sections with poly(A)-tailed antibody-derived tags followed by *in situ* barcoding of both ADTs and mRNAs, enabling simultaneous spatial profiling of the whole transcriptome and approximately 200–300 proteins at a spatial resolution of ∼25 μm.^86^

Imaging-based multimodal approaches achieve higher spatial resolution by combining multiplex RNA imaging with antibody-based protein detection. The 10× Genomics Xenium platform enables spatial co-profiling of RNA and proteins using targeted panels of up to 480 genes and 27 proteins, primarily designed for immune and tumor microenvironment studies. Vizgen’s MERSCOPE platform, based on MERFISH technology, allows simultaneous spatial profiling of hundreds to thousands of RNA transcripts alongside up to six proteins using oligonucleotide-conjugated secondary antibodies. SMI, implemented in the CosMx platform, provides detection of up to 1,000 RNA targets together with up to 64 proteins at subcellular resolution through iterative hybridization of fluorescent molecular barcodes.^87^ By integrating transcriptomic and proteomic measurements within the same spatial framework, these multimodal technologies provide a more comprehensive view of tissue organization, cellular phenotypes, and molecular interactions in complex biological systems.

#### ST and spatial epigenomics

Co-profiling of spatial gene expression and histone modifications or chromatin accessibility has been made possible through technologies motivated by DBiT-seq. MISAR-seq simultaneously profiles spatial gene expression and chromatin accessibility by combining principles from DBiT-seq and SHARE-seq,^88^ using a 2,500-pixel microfluidic grid (50 μm pixel resolution) design where channel-specific DNA barcodes are flowed through two perpendicular channels to spatially index Tn5-labeled open chromatin regions and reverse-transcribed mRNAs.^89^ The technology has been demonstrated on the fetal mouse brain to study cell fate determination and spatiotemporal regulatory dynamics during development; however, according to the authors, MISAR-seq is compatible with a wide range of tissue types. Similarly, to further investigate the epigenetic mechanisms underlying spatially resolved gene expression regulation, genome-wide co-mapping of the epigenome and transcriptome has been developed through spatial ATAC-RNA-seq at near single-cell resolution (20 μm pixel microfluidic channel array chip). These methods enable co-profiling of chromatin accessibility or histone modifications (e.g., H3K27me3, H3K27ac, and H3K4me3) alongside mRNA expression at cellular resolution via deterministic co-barcoding. By integrating the chemistry of spatial ATAC-seq or CUT&Tag with ST, these approaches provide comprehensive insights into the spatial coordination of epigenetic regulation and transcription. These technologies have been demonstrated in embryonic and juvenile mouse brains and adult human hippocampus to dissect the dynamic interplay between chromatin states and gene expression during development.^90^^,^^91^ Table 1 provides a summary of all the above strategies.

**Table 1:** Comparison of spatial omics technologies

Platform	Modality	Resolution	Readout	Throughput	Sample	H&E
ST (original)	T	100 μm	seq	WGE	FF	yes
10× Visium	T	55 μm	seq	WGE	FF/FFPE	yes
10× Visium HD	T	2 μm	seq	WGE	FFPE	yes
Slide-seq	T	10 μm	seq	WGE	FF	no
Slide-seq V2	T	10 μm	seq	WGE	FF	no
HDST	T	2 μm	seq	WGE	FF	yes
Curio Seeker	T	10 μm	seq	WGE	FF	no
Curio Trekker	T	single-nucleus	seq	WGE	FF	no
seqFISH/seqFISH+	T	subcellular	Img	up to 10,000 genes	FF	no
MERFISH/Merscope	T	100 nm	Img	up to 1,000 genes	FF/FFPE	no
CosMx (SMI RNA)	T	subcellular	Img	∼19,000 genes	FF/FFPE	no
Xenium (ISS)	T	subcellular	Img	up to 5,000 genes	FF/FFPE	yes
IMC	P	1 μm	MS	∼40 proteins	FF/FFPE	no
MIBI-TOF	P	260–1,000 nm	MS	∼40–100 proteins	FF/FFPE	no
CODEX	P	subcellular	Img	∼60 proteins	FF/FFPE	no
t-CyCIF/CycIF	P	subcellular	Img	20–60 proteins	FF/FFPE	no
MxIF	P	subcellular	Img	20–60 proteins	FF/FFPE	no
4i	P	subcellular	Img	>40 proteins	FF	no
IBEX	P	subcellular	Img	>65 proteins	FF/FFPE	no
DVP	P	single-cell	MS	>1,000 proteins	FF/FFPE	yes
Lunaphore COMET	P	subcellular	Img	∼40 proteins	FF/FFPE	no
GeoMx DSP	P	ROI-level	seq	∼40–100 proteins	FF/FFPE	yes
Spatial ATAC-seq	E	∼20 μm	seq	genome-wide	FF	no
Spatial CUT&Tag	E	∼20 μm	seq	genome-wide	FF	no
Chromatin tracing	E (3D)	30 nm	Img	targeted loci	FF	no
MALDI-MSI	M	5–50 μm	MS	hundreds of metabolites	FF/FFPE	no
DESI-MSI	M	50–200 μm	MS	hundreds of metabolites	FF	no
Visium CytAssist	T + P	55 μm	seq	WGE + 35 proteins	FF/FFPE	yes
SPOTS	T + P	55 μm	seq	WGE + >30 proteins	FF	yes
SM-Omics	T + P	100 μm	seq	WGE + 6 proteins	FF	yes
Spatial-CITE-seq	T + P	25 μm	seq	WGE + 200–300 proteins	FF	no
Xenium (multimodal)	T + P	subcellular	Img	480 genes + 27 proteins	FF/FFPE	yes
MERSCOPE (multimodal)	T + P	100 nm	Img	100–1,000 genes + 6 proteins	FF/FFPE	no
CosMx (multimodal)	T + P	subcellular	Img	1,000 genes + 64 proteins	FF/FFPE	no
MISAR-seq	T + E	50 μm	seq	WGE + chromatin access	FF	no
Spatial ATAC-RNA-seq	T + E	20 μm	seq	WGE + chromatin access	FF	no

T, transcriptomics; P, proteomics; E, epigenomics; M, metabolomics; Seq, sequencing-based; Img, imaging-based; MSI, mass spectrometry imaging; WGE, whole-genome expression; FFPE, formalin-fixed paraffin-embedded; and FF, fresh-frozen. Top: single-modality platforms and bottom: co-profiling platforms.

## Overview of multimodal spatial omics data integration strategies

There is a wide range of algorithms and strategies for integrating spatial omics modalities, and the choice of approach often depends on the specific acquisition strategy and the downstream computational task (Box 1). These methods can be conceptualized along two axes: the integration strategy and the fusion strategy. According to Argelaguet et al.^92^ and based on the experimental design, integration can be classified as horizontal, vertical, and diagonal. In multimodal spatial omics, horizontal integration refers to measuring the same modality across different donors, replicates, or experimental conditions. In this case, the common dimensions, or anchor, are the shared features, and alignment is performed between samples to enable comparative analysis across different conditions or samples. Vertical integration occurs when different spatial modalities (e.g., ST and spatial epigenomics) are measured either on the same tissue slice through spatial co-profiling or on serial slices with subsequent co-registration (Box 1). In this scenario, the anchor is the spatial spot, allowing cross-modal analysis across tissue and spatial niches. Diagonal integration is used when there is no anchor between the different datasets, for example, when integrating ST from one tissue sample with spatial proteomics from the same tissue type but from a different donor. In this case, a common computational strategy is to infer a shared latent space that captures correspondences between the different spatial layers.

**Figure 2:**
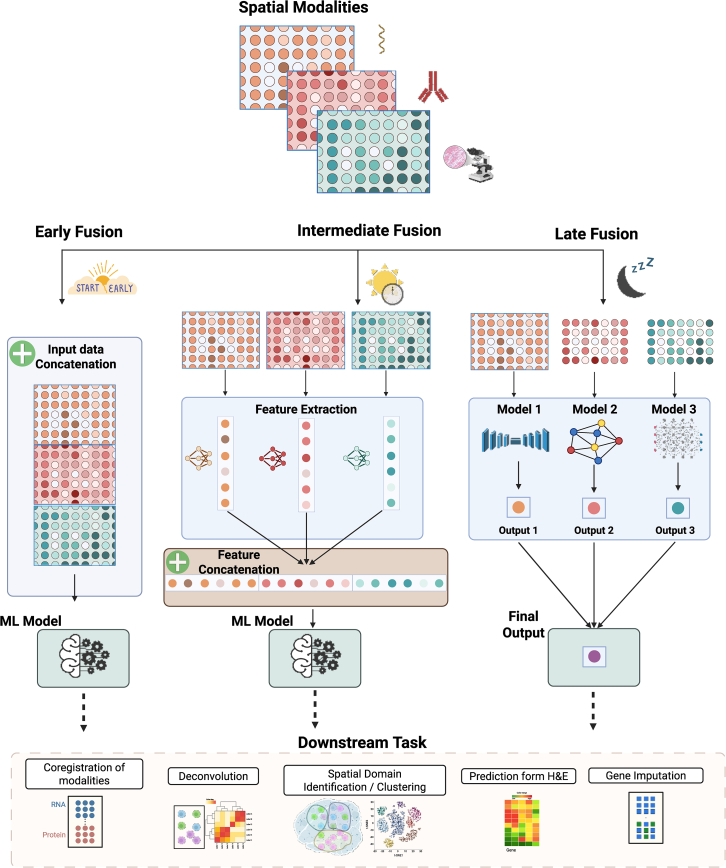
Fusion strategies for multimodal spatial omics integration Early fusion: modality-specific features are concatenated into a single input matrix before model processing. Intermediate fusion: each modality is encoded independently into latent representations, which are then combined for downstream tasks. Late fusion: separate models process each modality and predictions are aggregated at the decision level. Bottom illustrates the key downstream computational tasks: co-registration, deconvolution, spatial domain identification, molecular prediction from H&E, and generative modeling.

The second axis concerns the fusion strategy (Figure 2). Whereas the integration strategy defines the conceptual framework for aligning multiple modalities along a common dimension, the fusion strategy describes when and how information from each modality is computationally combined. In early fusion, the modality-specific features are concatenated into a single matrix before being fed into the final integration model. Early fusion, also known as feature-level fusion, is simple and straightforward, as a single model is used to learn a joint representation of the multimodal data. Moreover, in deep learning approaches, its lower architectural complexity makes it a popular and convenient choice. However, early fusion also introduces important limitations. Spatial multimodal omics datasets are inherently heterogeneous and span different scales, noise characteristics, statistical distributions, and data structures (e.g., histology images versus counts-based ST). Effective modality-specific preprocessing and normalization are therefore essential and can strongly influence downstream model performance. Moreover, by simply concatenating all modalities into a single input, the model loses information about modality identity, hindering its ability to infer complex or non-linear cross-modal interactions.

In late fusion, or decision-level fusion, each modality is processed independently through its own model, and integration occurs only at the prediction level, where the individual model outputs are combined into a final decision. This is similar to the approach taken by ensemble methods. Common aggregation techniques include majority voting, averaging probabilities, or weighted averaging. The modularity of the late fusion approach offers a flexible way of integrating multimodal spatial omics data, allowing different model architectures—and therefore diverse data types—to be used for each modality. Furthermore, new modalities can easily be added to the pipeline without requiring modification or retraining of the existing modality-specific models. Missing modalities are a common phenomenon observed in spatial multimodal omics; for example, when integrating ST and spatial proteomics from serial tissue sections, some samples may lack one of the modalities. Late fusion can handle such scenarios because the predictions are generated independently by each model. However, if cross-modal independence cannot be assumed, the “first model, then integrate” strategy can overlook cross-modal interactions, which are often essential for downstream analysis and biological interpretation (Figure 2).

Intermediate fusion, also called representation-level fusion, is a hybrid of early and late fusion that aims to learn a joint representation of the different spatial omics modalities. In this approach, each modality is first processed independently to extract modality-specific latent representations, which are then combined into a joint representation matrix that is fed into the final model for downstream tasks. Methods for merging the individual representations include simple concatenation, element-wise operations (summation, averaging, or multiplication), as well as more advanced approaches based on attention mechanisms. Modality-specific preprocessing allows the use of tailored models for each modality, facilitating the extraction of high-quality latent features that capture within-modality correlations. These features, in turn, enable the final model to learn more complex and non-linear interactions both within and across modalities. A key limitation of intermediate fusion is modality imbalance, where features from one modality might dominate the joint representation, leading to biased embeddings. Approaches such as modality-specific weighting can mitigate these effects and help the model to perform more balanced predictions (Figure 2).

One of the key challenges in integrating data from different spatial technologies (via adjacent tissue slices) lies in the mismatch of the spatial resolution. For example, imaging-based spatial proteomics technologies achieve subcellular resolution, whereas sequencing-based ST technologies, such as Visium, and MSI-based spatial metabolomics typically operate at multicellular resolution. At these scales, each spot or pixel often captures signals from multiple cells. To enable integration across these modalities, measurements from higher-resolution platforms are commonly aggregated to match the spatial scale of the lower-resolution data, leading to the loss of subcellular information. In addition, discrepancies in the alignment and registration of adjacent tissue sections can further obscure cell-type-specific populations, resulting in reduced accuracy of cell-type assignment.

Alternatively, super-resolution approaches aim to refine the lower-resolution measurements by inferring gene expression profiles and cell types at locations between the spatial spots, thereby offering single-cell resolution^93^ and allowing integration with higher-resolution modalities. However, as these methods rely on predictive inference rather than direct measurement, they introduce additional sources of error that may propagate through downstream analyses and lead to inaccurate biological interpretations.

Together, these limitations highlight the trade-off between resolution and reliability, suggesting that co-profiling technologies provide the most direct and reliable basis for integration.

## Computational frameworks for multimodal spatial omics integration

**Figure 3:**
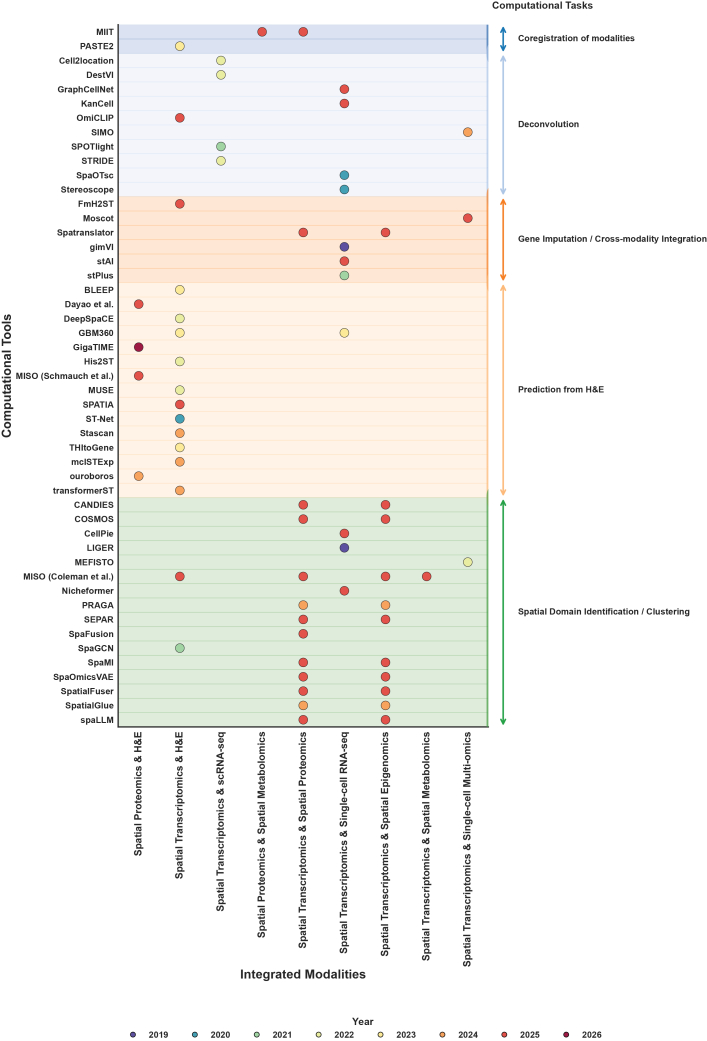
Landscape of computational tools for multimodal spatial omics integration Published methods mapped by modality combinations and downstream tasks (co-registration, deconvolution, spatial domain identification, molecular prediction from H&E, and generative modeling). The timeline highlights accelerated development from 2022 onwards. Spatial transcriptomics—H&E integration is the most mature area, while epigenomics, metabolomics, and multimodal proteomics integration remain underexplored. Note: some tools support multiple tasks; ordering and shading reflect the primary task.

The integration strategies outlined above provide a conceptual framework for aligning multimodal spatial omics and imaging datasets across experimental designs, resolutions, and feature spaces. However, translating these strategies into practical tools requires concrete computational implementations. Figure 3 provides an overview of the rapidly expanding ecosystem of computational tools, summarizing the modality combinations they integrate and the downstream computational tasks, as defined in Box 1.

In the following subsections, we review the major classes of computational frameworks for multimodal spatial omics integration, organizing methods by their underlying algorithmic principles while highlighting how each method addresses key computational tasks in spatial omics analysis (Box 1; Figure 3; Table 3).

**Table 3:** Summary of computational tools for multimodal spatial omics integration

Tool	Algorithm	Fusion	Language	Code availability
MIIT	geometry-driven co-registration	N/A	Py	https://github.com/mwess/miit
PASTE2	fused Gromov-Wasserstein OT	N/A	Py	https://github.com/raphael-group/paste2
Cell2location	hierarchical Bayesian model	I	Py	https://github.com/BayraktarLab/cell2location
DestVI	variational inference	I	Py	https://github.com/scverse/scvi-tools
OmiCLIP	transformer/ViT (contrastive)	I	Py	https://github.com/GuangyuWangLab2021/Loki
SIMO	optimal transport + graph learning	I	Py	https://github.com/ZJUFanLab/SIMO
SPOTlight	seeded NMF + NNLS	I	R	https://github.com/MarcElosua/SPOTlight
STRIDE	topic modeling	I	Py	https://github.com/DongqingSun96/STRIDE
SpaOTsc	structured OT mapping	I	Py	https://github.com/zcang/SpaOTsc
Stereoscope	probabilistic	I	Py	https://github.com/almaan/stereoscope
FmH2ST	CNN + transformer + GAT	N/A	–	–
Moscot	Fused Gromov-Wasserstein OT	I	Py	https://github.com/theislab/moscot
SpaTranslator	GNN + generative learning	I	Py	https://github.com/donghongyu2020/SpaTranslator
gimVI	probabilistic VAE	I	Py	https://github.com/scverse/scvi-tools
stAI	autoencoder (maximum mean discrepancy-based)	I	Py	https://github.com/gszou99/stAI
stPlus	autoencoder	I	Py	https://github.com/xy-chen16/stPlus
BLEEP	pre-trained ResNet50 + FCN	N/A	Py	https://github.com/bowang-lab/BLEEP
Dayao et al.	CNN	I	Py	https://github.com/mdayao/hist-prot-integration
DeepSpaCE	CNN	N/A	Py, R	https://github.com/tmonjo/DeepSpaCE
GBM360	CNN	N/A	Py	https://github.com/gevaertlab/GBM360
Hist2ST	ConvMixer + transformer + GNN	I	Py	https://github.com/biomed-AI/Hist2ST
MISO (Schmauch)	ViT + local attention MIL	N/A	Py	https://github.com/owkin/miso_code
MUSE	autoencoder	I	Py	https://github.com/AltschulerWu-Lab/MUSE
SPATIA	hierarchical cross-attention + OT	I	Py	–
ST-Net	CNN	N/A	Py	https://github.com/bryanhe/ST-Net
STASCAN	CNN	N/A	Py	https://github.com/AbbyWY/STASCAN
THItoGene	ViT/GAT	I	Py	https://github.com/yrjia1015/THItoGene
mclSTExp	transformer	I	Py	https://github.com/ZhicengShi/mclSTExp
Ouroboros	GAN	N/A	Py	https://github.com/Srijay/Ouroboros
TransformerST	ViT + GCN + graph transformer	I	Py	https://github.com/Zhaocy-Research/TransformerST
CANDIES	conditional diffusion + contrastive	I	Py	https://github.com/YeLiu-Lab/CANDIES
COSMOS	GCN + deep graph ifomax	I	Py	https://github.com/Lin-Xu-lab/COSMOS
CellPie	joint NMF	I	Py	https://github.com/ManchesterBioinference/CellPie
LIGER	integrative NMF (iNMF)	I	R, Py	https://github.com/welch-lab/liger
MEFISTO	Bayesian factor analysis + GP	I	R, Py	https://github.com/bioFAM/MOFA2
MISO (Coleman)	adjacency-graph GNN	I	Py	https://github.com/kpcoleman/miso
Nicheformer	transformer	I	Py	https://github.com/theislab/nicheformer
PRAGA	omics-aware graph + contrastive	I	Py	https://github.com/Xubin-s-Lab/PRAGA
SEPAR	spatial metagene decomposition	E	Py	https://github.com/zerovain/SEPAR
SpaFusion	graph autoencoder + transformer	I	Py	https://github.com/polarisChen/SpaFusion
SpaGCN	GCN	I	Py	https://github.com/jianhuupenn/SpaGCN
SpaOmicsVAE	GNN + VAE	I	Py	https://github.com/ZhiWeiZhang0336/SpaOmicsVAE
SpatialFuser	multi-head graph attention	I	Py	https://github.com/liwz-lab/SpatialFuser
SpatialGlue	graph attention network	I	Py	https://github.com/JinmiaoChenLab/SpatialGlue
GraphCellNet	KAN + GCN	I	Py	https://github.com/DaiRuoYan-123/GraphCellNet-
KanCell	Kolmogorov-Arnold networks	I	Py	https://github.com/DaiRuoYan-123/KanCell-main
GigaTIME	NestedUNet (CNN-based)	N/A	Py	https://github.com/prov-gigatime/GigaTIME
SpaMI	graph autoencoder	I	Py	https://github.com/Gaocongqiang/SpaMI
novoSpaRc	structured OT	N/A	Py	https://github.com/rajewsky-lab/novosparc
spaLLM	scGPT + GNN + attention	I	Py	https://github.com/liiilongyi/spaLLM

Algorithm, fusion strategy (E, early; I, intermediate; L, late; and N/A, not applicable), programming language (Py, Python), and code availability for each tool reviewed in this work.

### Probabilistic and statistical inference methods

**Figure 4:**
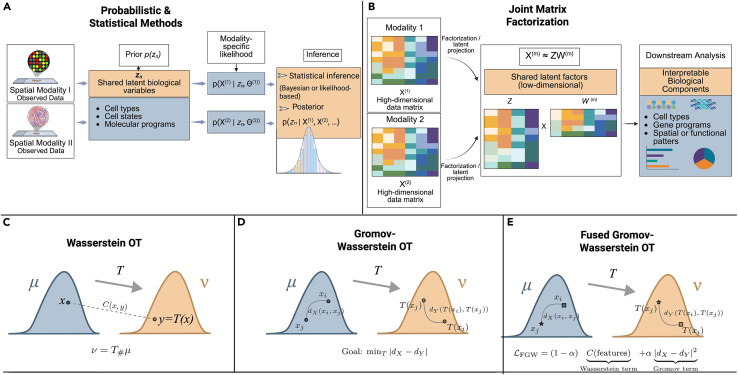
Computational frameworks for multimodal spatial omics integration (A) Probabilistic inference: shared latent variables *z*_*n*_ at each location are inferred from multimodal observations via modality-specific likelihoods *p*(*X*^(*m*)^∣*z*_*n*_,Θ^(*m*)^) and prior distributions *p*(*z*_*n*_∣Φ). (B) Matrix factorization: data matrices are decomposed into shared factors *Z* and modality-specific loadings *W*^(*m*)^ such that *X*^(*m*)^≈*ZW*^(*m*)^. (C) Wasserstein OT finds the minimal-cost transport plan to align distributions in a common feature space. (D) Gromov-Wasserstein OT aligns datasets across different feature spaces by preserving pairwise distance structure. (E) Fused Gromov-Wasserstein OT combines feature-level (Wasserstein) and structure-preserving (Gromov) costs, weighted by *α*.

Multimodal spatial omics integration can be formulated as a probabilistic inference problem, in which measurements from multiple data modalities are assumed to arise from shared latent variables that capture the underlying biological structure.^94^^,^^95^ In this setting, a latent variable *z*_*n*_ is associated with each spatial location or spot *n* and represents shared biological properties such as cell-type composition, cell states, or low-dimensional transcriptional programs (Figure 4A). Each modality *m*∈{1, …,*M*} is described by a modality-specific likelihood that captures its distinct measurement process, noise characteristics, and data distribution. Let ${\left\{X^{\left(m\right)}\right\}}_{m=1}^{M}$ denote multimodal spatial measurements (e.g., ST gene expression counts and spatial proteomics protein intensities). The generative model is defined as

(Equation 1) $$X_{n}^{\left(m\right)}∼p\left(X_{n}^{\left(m\right)}|z_{n},\Theta^{\left(m\right)}\right),z_{n}∼p\left(z_{n}|\Phi \right)\text{,}$$

where *z*_*n*_ represents latent variables that encode the underlying biological state at spatial location *n* (e.g., cell-type composition or latent factors). The parameters Θ^(*m*)^ denote modality-specific generative parameters, such as latent factor loadings linking *z*_*n*_ to observed gene or protein measurements, as well as additional parameters governing the observation model (e.g., dispersion or noise parameters). These parameters may be learned directly from the data or informed by external reference profiles, such as cell-type specific expression or protein abundance signatures. The prior over latent variables is specified by Φ.

The multimodal integration task then corresponds to estimating the posterior distribution

(Equation 2) $$p\left(z_{n}|X_{n}^{\left(1\right)},\ldots ,X_{n}^{\left(M\right)}\right)\text{,}$$

which is generally intractable and therefore approximated using variational inference or related Bayesian approximation techniques.

Several spatial deconvolution tools adopt this probabilistic formulation to integrate ST with scRNA-seq references. Stereoscope^96^ models spatial gene expression as a mixture of reference-derived cell-type profiles under a negative binomial (NB) likelihood, enabling estimation of cell-type proportions together with uncertainty. Cell2location^97^ extends this approach using a hierarchical Bayesian model that explicitly accounts for technical effects and overdispersion, improving robustness and sensitivity, particularly for rare cell types. DestVI further generalizes probabilistic deconvolution by introducing continuous latent variables to model within-cell-type state variation, combining Bayesian inference with neural parameterization in a variational framework.^98^

Hybrid approaches combine probabilistic inference with deep learning for spatial prediction tasks. MUSE integrates histological images (H&E) with ST using a framework that includes probabilistic components alongside neural networks, enabling prediction of gene expression from tissue morphology while retaining a distributional view of the data.^99^ Similarly, CANDIES incorporates probabilistic inference within a broader deep learning-based multi-omics integration pipeline, allowing uncertainty-aware spatial domain identification while integrating ST with spatial epigenomic or proteomic modalities.^100^

More broadly, these spatial probabilistic models are conceptually related to classical Bayesian multi-view approaches such as multiple dataset integration (MDI)^94^ and Bayesian factor analysis models, including MOFA and its spatially structured extension MEFISTO.^5^^,^^95^^,^^101^ The latter introduces Gaussian process priors that allow latent factors to vary smoothly across spatial coordinates.

Probabilistic and statistical inference methods provide a rigorous and interpretable framework for multimodal spatial omics integration, enabling principled deconvolution, cross-modal alignment, and uncertainty quantification across heterogeneous spatial datasets.

### Matrix factorization and latent variable models

Matrix factorization and latent variable models integrate multimodal spatial omics data by decomposing high-dimensional observations into low-rank representations that capture shared biological structure across modalities. These methods assume that spatial measurements from different assays can be explained by a small number of latent factors corresponding to cell types, gene programs, or spatial patterns, providing an interpretable and computationally efficient framework for integration.

A common formulation is the joint or integrative non-negative matrix factorization (NMF), where each modality-specific data matrix $X^{\left(m\right)}\in R^{N\times G_{m}}$ is approximated as

(Equation 3) $$X^{\left(m\right)}\approx ZW^{\left(m\right)}\text{,}$$

with $Z\in R^{N\times K}$ representing the shared latent factors across spatial locations and $W^{\left(m\right)}\in R^{K\times G_{m}}$ capturing modality-specific loadings (Figure 4B). Non-negativity constraints encourage additive and biologically interpretable factors. CellPie applies this joint NMF formulation to ST, enabling spatial domain identification by jointly modeling ST gene expression and morphological features derived from paired H&E images.^102^ Similarly, LIGER uses an integrative NMF approach to jointly factorize multiple unpaired datasets into shared and modality-specific factors.^103^ Latent factor formulations are used in SIMO and SEPAR, which integrate ST with single-cell or spatial multi-omics data to jointly perform spatial domain identification and, in some cases, deconvolution.^104^^,^^105^ SPOTlight adopts a related NMF-based strategy but incorporates prior knowledge through marker genes from scRNA-seq datasets, yielding seeded latent topics that correspond directly to known cell types.^106^

Several recent methods combine matrix factorization with deep learning components. STRIDE and SpaFusion incorporate NMF-like latent representations together with neural networks to improve robustness and capture non-linear structure, while still retaining interpretable factor-based decompositions.^107^^,^^108^ COSMOS similarly blends latent variable modeling with deep learning to integrate ST with spatial proteomics or epigenomics for spatial domain identification and deconvolution.^109^

Matrix factorization and latent variable models provide an interpretable middle ground between purely probabilistic inference and fully deep learning–based approaches. By explicitly modeling shared and modality-specific factors, they are well suited for exploratory multimodal spatial analysis, spatial deconvolution, and identification of dominant biological programs across heterogeneous spatial omics datasets.

### OT and geometric alignment

OT and geometric approaches tackle spatial multi-omics integration from a distance and structure-preserving perspective. In this paradigm, spatial locations, cells, and molecular profiles are represented as empirical measures embedded in a metric space, and cross-modal or cross-sample alignment is formulated as an optimization problem that minimizes transport cost under a prescribed cost function. This geometric view is particularly attractive for spatial omics data, where spatial coordinates, tissue architecture, and neighborhood relations carry as much information as the expression profiles themselves.

Current methods based on OT rely on the Wasserstein distance to quantify differences between distributions in a common feature space, interpreting this difference as the minimal effort required to transform one distribution into the other.^110^ For example (Figure 4C), OT can relate a group of single cells to a set of spatial spots by estimating a transport plan *π*, which can be interpreted as a weight (or probability) matrix linking cells to spots. The plan is computed to minimize a cost that combines transcriptomic dissimilarity and spatial proximity, while ensuring that the total mass assigned to cells and spots remains consistent.

At present, most Wasserstein OT methods are mainly used for *in silico* cell-to-space mapping (mapping dissociated single cells to spatial coordinates). For example, SpaOTsc uses structured OT to couple scRNA-seq with spatial data and infer spatially informed cell-cell distances/communication,^111^ and novoSpaRc learns a probabilistic cell-to-location assignment for spatial reconstruction.^112^

However, many spatial multi-omics tasks require aligning datasets that are represented in distinct feature spaces, such as transcriptomic and proteomic measurements, or gene expression and histology-derived features. Gromov-Wasserstein (GW) OT and its fused variants address this setting by matching the internal relational structure of two datasets rather than their raw coordinates.^110^ As illustrated in Figure 4D, instead of directly penalizing the distances between individual points, GW minimizes discrepancies between pairwise distance matrices, encouraging correspondences that preserve local similarity structure and underlying topology. Figure 4E illustrates that fused GW (FGW) further incorporates feature-level costs, allowing simultaneous alignment of expression similarities and spatial or morphological distances.

By encoding spatial coordinates, neighborhood graphs, or anatomical annotations into the cost tensor, GW-based methods can preserve tissue continuity, boundaries, and layered organization during integration, and are naturally suited to handling resolution mismatches, multi-section co-registration, and cross-modal alignment. However, the current body of GW/FGW-driven spatial integration work remains relatively small, with representative examples including PASTE/PASTE2, which use fused Gromov-Wasserstein OT to align adjacent ST sections and integrate multiple slices into a consensus (center) slice or support 3D stacking and reconstruction^6^^,^^113^; Moscot, a scalable OT framework that supports FGW-based multimodal and spatial dataset alignment^114^; and SIMO, which takes ST and multi-omics single-cell datasets as input and performs FGW-OT-based probabilistic cell-spot alignment for spatial mapping and downstream analyses.^104^

In parallel to OT-based alignment, geometric alignment toolsets integrate spatial multi-omics via explicit cross-section co-registration and resolution harmonization. For example, multi-omics imaging integration toolset (MIIT) first performs histology-guided nonrigid section registration (using GreedyFHist) to align serial sections, then maps ST and MSI onto a common grid and aggregates MSI pixels into each corresponding ST spot weighted by the overlap area, thereby fusing ST and MSI data. Notably, MIIT demonstrates spatial metabolomics integration as its primary use case, providing one of the few end-to-end pipelines for combining MSI with ST. This corresponds to a deterministic geometry-driven matching, rather than learning a transport plan by optimizing an OT objective.^115^

### Deep learning and foundation models

Deep learning models now play a central role in multimodal spatial omics integration, providing flexible neural architectures that can capture the heterogeneous, non-linear, and spatially structured relationships inherent to these complex and high-dimensional datasets. Deep learning has recently undergone a major paradigm shift with the development of foundation models. Trained on vast, heterogeneous datasets using self-supervised learning, these models acquire general-purpose representations that can be rapidly adapted or fine-tuned to a wide range of downstream tasks—including multimodal spatial omics—using only modest amounts of task-specific data.

### CNN models

**Figure 5:**
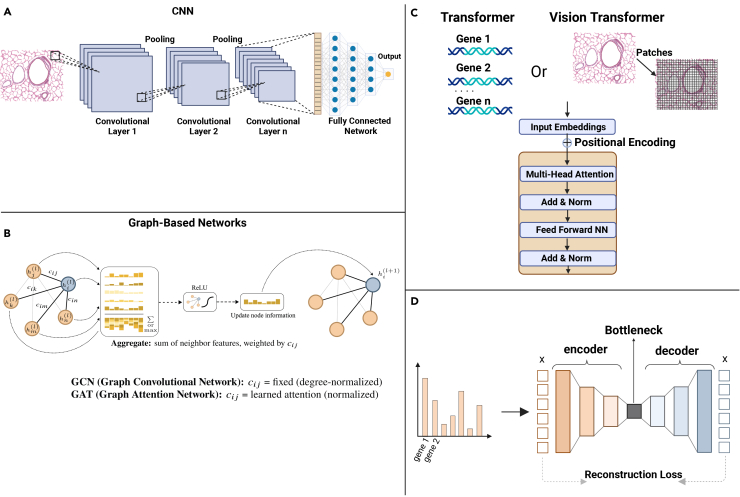
Deep learning architectures for multimodal spatial omics (A) CNNs extract hierarchical features from histology patches via convolutional and pooling layers. (B) Graph neural networks represent spatial locations as nodes with edges encoding adjacency; GCNs use fixed aggregation weights, while GATs learn adaptive attention weights. (C) Transformers apply self-attention to capture long-range dependencies, treating genes or proteins as input tokens with positional encodings preserving spatial information. Multi-head attention computes pairwise relationships across the sequence, enabling discovery of coordinated gene programs. ViTs adjust this framework for histology images by partitioning images into patches, embedding each as a token, and applying transformer layers to capture global morphological context. (D) Autoencoders learn a compact representation of high-dimensional spatial omics data by encoding the inputs into a low-dimensional bottleneck and reconstructing them back through a decoder. The reconstruction loss encourages the latent space to preserve biologically meaningful structure, making autoencoders useful for multimodal spatial omics data analysis, integration, and gene imputation.

Convolutional neural networks (CNNs) have been widely used in multimodal spatial omics integration as versatile encoders of histological images, serving two primary roles: directly as backbone architectures for predicting spatial omics profiles from H&E images, and indirectly as feature extractors that provide morphology-informed inputs to downstream integration architectures. CNNs are hierarchical models typically composed of stacked convolutional layers that progressively extract increasingly abstract features from input images.^116^^,^^117^ Early convolutional layers extract local features (such as texture) through learned filters, while deeper layers combine these early features into more abstract, high-level features, allowing the model to learn more complex tissue structures.^116^^,^^118^ Spatial dimensionality of the input images is reduced through pooling operations, improving computational efficiency while retaining important features. The resulting feature maps are transformed into vector representations and passed to a fully connected layer that produces task-specific outputs^116^^,^^118^^,^^119^ (Figure 5A). During training, model parameters are learned through gradient-based optimization, with backpropagation used to update the weights.^116^^,^^119^

Among the earliest methods, ST-Net^120^ uses a CNN-based architecture to learn mappings between histology image patches and spatial gene expression, enabling the prediction of spatial gene expression profiles from histology. STASCAN predicts spatial cell-type distributions, allowing cell-type composition to be inferred in regions not directly assayed by low-resolution ST. This enables both spatial resolution enhancement and 3D map construction using imputation on consecutive tissue sections.^121^ At the gene level, analogous CNN-based approaches such as DeepSpaCE predict enhanced spatial gene expression profiles directly from H&E images and enable the imputation of gene expression maps between adjacent tissue sections.^122^ Building on these predictive frameworks, Zheng et al.^123^ developed a CNN-based model that integrates paired glioblastoma histology images, 10× Visium ST data and clinical outcome to predict the spatial distribution of immune cell types and tumor aggressiveness from unseen H&E images.

Beyond CNNs, in the field of spatial proteomics, an alternative generative-predictive approach is provided by Ouroboros, a tool based on generative adversarial neural networks (GANs), that enables simultaneous prediction of spatial proteomics profiles from H&E images as well as H&E image generation from spatial protein profiles.^124^ These methods enable cost-effective spatial molecular profiling as well as in silico perturbation experiments.

#### Autoencoders and contrastive learning

Autoencoders (AE) and variational autoencoders (VAE) enable compact representation learning by encoding high-dimensional spatial omics data into a low-dimensional latent space and reconstructing the input through a decoder, as illustrated in Figure 5D. The learned representations capture biologically meaningful structure because they are optimized via a reconstruction objective that preserves key patterns in the data. VAEs further extend this framework by imposing a probabilistic structure on the latent space through a variational objective (e.g., KL divergence regularization), which encourages smooth, continuous embeddings. The power of latent spaces in AEs is well-suited for multimodal integration, as demonstrated in MUSE, where modality-specific embeddings from ST and images are fused into a shared feature space and refined using reconstruction and self-supervised objectives.^99^ SpaOmicsVAE proposes an architecture to generate a unified representation of any two desired spatial omics data, which are encoded through dual graph neural networks (GNNs). It uses the power of VAEs to make the framework more generalizable and to preserve proper distribution within the latent space using the Kullback-Leibler (KL) divergence loss alongside the reconstruction loss.^125^ In the field of spatial omics, unmeasured gene expression imputation is a challenging problem that has been recently addressed using AE architectures. stPlus predicts expression of unmeasured genes by learning the joint embedding between scRNA-seq (reference data) and spatial data while leveraging genes unique to the reference. It then predicts unmeasured gene expression via weighted k-nearest neighbors in the learned latent space.^126^ Along the same line, gimVI introduces a generative VAE model to impute missing genes from unpaired scRNA-seq and ST by first learning a shared latent cell-state space with modality-specific likelihoods. A shared neural-network decoder maps the latent variable to gene expression proportions. After training, unmeasured genes in spatial cells are imputed by decoding their inferred latent state.^127^ stAI extends the framework of joint-embedding approaches by introducing a dual encoder-decoder architecture that performs both gene imputation and cell-type annotation. stAI first aligns the two datasets by maximum mean discrepancy-based latent alignment and improves the imputation using supervised calibration genes and extra novel loss functions such as gene consistency loss.^128^

In the field of multimodal data analysis, contrastive learning has been an emerging area of research. Contrastive learning focuses on creating a shared embedding space between paired data modalities by maximizing the similarity between positive pairs, meaning two modalities from the same spot, cluster, or sample, while minimizing the similarity between negative pairs using similarity-based contrastive loss. Inspired by CLIP,^129^ contrastive learning is employed in mclSTExp and BLEEP to learn a joint latent space aligning paired H&E images and ST, enabling a new H&E image to be embedded and its gene expression to be inferred via similarity in the latent space.^130^^,^^131^ Another approach is to utilize a multimodal contrastive loss function to map H&E and spatial proteomics (CODEX) measurements into a joint representation space for cell-type annotation.^132^ In this setup, positive pairs consist of H&E-CODEX patches from the same cell, while negative pairs include all other combinations. The contrastive loss encourages embeddings of positive pairs to be close to each other. Recently, SpaMI^133^ introduced its idea for integrating multimodal spatial omics by training graph autoencoders in a contrastive manner. To capture modality-specific embeddings, each omics is encoded as a graph both in its original and corrupted versions to create positive and negative pair embeddings, which are optimized using a contrastive loss. The final representation space is obtained through an adaptive attention mechanism between each modality-specific embedding.

#### Graph-based models

Within multimodal spatial omics integration, a major branch of deep learning work focuses on GNNs. In these methods, tissue locations or cells are represented as nodes in a graph, and edges are used to encode spatial adjacency, histology-derived similarity, or neighborhood relationships at the molecular level. Graph convolutional networks (GCNs), one of the simplest and most widely used GNN architectures, update node representations as illustrated in Figure 5B by linearly transforming and aggregating the embeddings of neighboring nodes.^134^ In spatial multi-omics, GCN-based architectures, exemplified by methods such as SpaGCN and GraphST, have been widely used to integrate gene expression, spatial location, and histology to identify spatial domains and spatially variable genes.^135^^,^^136^ GraphCellNet uses a GCN architecture for spatial domain identification, while deconvolution is performed through an encoder-decoder architecture that incorporates a Kolmogorov-Arnold network (KAN) layer in the encoder to model non-linear feature relationships. The decoder further incorporates a self-attention mechanism that enables global, context-dependent interactions that allow the model to capture complex expression patterns and functional dependencies. For spatial domain identification, the method better resolves ambiguous cell boundaries that are difficult to delineate, while maintaining computational efficiency.^137^ Another method, based on KAN, KanCell, integrates ST and scRNA-seq data to deconvolve cellular heterogeneity. KanCell incorporates self-attention to capture long-distance dependencies in high-dimensional ST data and offers enhanced modeling of complex multidimensional relationships while providing efficient feature representations and improved computational performance.^138^

As an extension of GCNs, graph attention networks (GATs) introduce learnable attention weights to adaptively modulate the contributions of different neighbors to node updates.^139^ In such models, node updates typically follow the scheme illustrated in Figure 5B, where the attention coefficients $c_{ij}^{\left(l\right)}$ are computed from pairwise interactions between neighboring nodes and can be extended to a multi-head attention mechanism. This enables GATs to focus on the most informative spatial or molecular neighbors and to better characterize heterogeneous microenvironments, sharp tissue boundaries, and directional cell-cell interactions. This attention-based design has been widely adopted in spatial multi-omics applications, with methods such as SpatialGlue^140^ and SpatialFuser^141^ exemplifying its use across tasks, including identification of spatial domains, expression denoising, and multi-sample integration, highlighting its clear advantages in representing complex spatial microenvironments. In addition to message-passing GNNs, there are also adjacency graph-centric approaches for multimodal feature extraction and clustering; MISO is one such framework, integrating multiple spatial modalities including ST, spatial proteomics, spatial epigenomics, spatial metabolomics, and paired histology images.^142^

Beyond pre-defined graphs with fixed connectivity, recent work has emphasized learning more adaptive graph structures and modality-specific contributions for paired spatial multi-omics measured on shared coordinates. PRAGA is designed for paired spatial multi-omics integration (such as ST + ATAC or ST + protein) by learning omics-aware graph representations and improving robustness to noisy measurements and unknown annotations, producing unified embeddings for downstream spatial domain discovery and related analyses.^143^ Similarly, COSMOS targets paired spatial multi-omics integration (e.g., transcriptomics with epigenomics/ATAC or proteomics) by jointly modeling each modality on graphs and combining them with learned modality contributions and contrastive objectives to obtain spatially consistent integrated representations.^109^

Graph models have also been used in cross-modal translation settings, where the goal is to infer an unmeasured spatial modality from an observed one. SpaTranslator performs cross-modal translation across spatial modalities, enabling the prediction of paired spatial multi-omics, notably translating between ST and spatial epigenomics as well as between ST and spatial proteomics, by leveraging neighborhood-aware graph encoders together with generative learning.^144^ In addition, graph-based designs are increasingly used for imaging-omics bridging; for example, FmH2ST predicts ST from histological images by combining image-derived features with spatial graph modeling to improve gene expression prediction and denoising.^145^

Graph autoencoders extend GNNs by optimizing reconstruction losses on the adjacency matrices to learn informative graph representations. SpaFusion utilized the power of this architecture to fuse the graph encodings of ST and proteomics and perform clustering on the unified representation.^108^

#### Transformer-based models

Originally introduced for natural language processing (NLP), the transformer architecture combines the self-attention mechanism with feedforward neural networks to model long-range dependencies and capture contextual relationships within sequential data^146^ (Figure 5C). In NLP, transformer-based models operate on a sequence of input tokens, where each token usually corresponds to a single word. These tokens are mapped to vector representations, known as embeddings, that encode semantic similarity. As transformer architectures are inherently agnostic to the order of the input tokens, positional embeddings are added to the input token embeddings to encode positional information within the sequence. Self-attention then computes pairwise similarity scores between these embeddings via scaled dot products, enabling the modeling of contextual relationships within the sequence (Figure 5C). These token-based abstractions can be generalized to omics applications such that each token represents a biologically meaningful unit, for example genes, proteins, or metabolites. Applying attention mechanisms to a sequence of omics tokens allows the transformer to learn context-dependent interactions (e.g., gene-gene or protein-protein relationships), facilitating the identification of coordinated biological programs, including gene regulatory networks. Building on this paradigm, spaLLM combines single-cell and spatial omics data by using scGPT, a pre-trained single-cell large language model (LLM), together with GNNs and multi-view attention aggregation to enhance spatial domain identification.^147^^,^^148^ Importantly, SpaLLM has been demonstrated across diverse spatial omics technologies, including MISAR-seq, 10× Visium, spatial-CITE-seq, and SPOTS.

As an alternative to CNNs, vision transformers (ViTs) extend the transformer framework to computer vision.^149^^,^^150^^,^^151^ Similarly to CNNs, ViTs act as visual encoders of images.^150^ In contrast to vanilla CNNs, ViTs capture global spatial context and long-range dependencies through the self-attention mechanism.^152^^,^^153^^,^^154^ Analogous to standard transformers, ViTs treat images as sequences by partitioning them into fixed-size, non-overlapping patches, each of which is flattened into a 1D vector and mapped to a high-dimensional embedding. To preserve spatial information, positional embeddings are added to these embeddings^152^^,^^153^ (Figure 5C). Global context and long-range dependencies between image patches are then learned through self-attention, followed by a feedforward network that applies non-linear transformations to each embedding.^152^^,^^153^^,^^154^

A series of methods that incorporate transformers and ViT components have been developed to predict spatial gene expression from histology images. These methods are typically implemented as hybrid models that combine transformer-based architectures with CNNs or graph-based models. TransformerST combines ViTs, graph transformers, and GCNs to enhance spatial gene expression resolution from 10× Visium data to the single-cell level, enabling improved clustering performance.^155^ Similarly, THItoGene adopts a hybrid framework that combines CNNs, ViTs, and GATs, while Hist2ST combines CNN, transformer, and GNN architectures for spatial gene expression prediction from histology images.^156^^,^^157^ Related hybrid architectures, such as MISO, leverage ViTs with local attention multiple instance learning to predict spatial gene expression from histology.^158^ Unlike the standard global self-attention, local attention in MISO is restricted to neighboring instances defined by the k-nearest neighbors based on Euclidean distance between the spatial locations, thereby reducing the computational burden. SPATIA is a multi-scale tool that fuses imaging-based ST gene expression across scales, from cellular to tissue level, into a unified embedding through hierarchical cross-attention mechanisms. SPATIA supports both predictive and generative tasks, including cell annotation, gene imputation, and image generation conditioned on gene expression. In addition, the authors have curated and released a large benchmarking dataset for imaging-based ST with one-to-one matched morphology and gene expression profiles, spanning multiple donors, tissues, and disease types.^159^

#### Multimodal foundation models

The development of multimodal foundation models for spatial omics is an emerging field. Nicheformer is a recent proof-of-concept transformer-based multimodal method pre-trained on large-scale human and mouse imaging-based ST and single-cell datasets, comprising over 110 million cells across diverse technologies.^160^ Nicheformer learns a joint representation of spatial and single-cell transcriptomics data through modality-aware contextual tokens, enabling in silico mapping of dissociated single-cell profiles into a spatially informed space. OmiCLIP is a dual-encoder image-transcriptomics foundation model trained on paired 10× Visium ST data and whole-slide images from 1,007 tissue samples spanning 32 organs. OmiCLIP supports a wide range of downstream tasks, including spatial gene expression prediction from unseen H&E images, ST-H&E image alignment, and cell-type deconvolution.^161^ OmiCLIP’s architecture consists of two modality-specific encoders: an image encoder based on a ViT and a transcriptomics encoder based on a causal-masked transformer. Whole-slide histology images are cropped into patches corresponding to the spatial resolution of 10× Visium spots and processed by the image encoder, while for each spot, the top 50 expressed genes, ranked by expression level, are converted into a tokenized gene sequence and passed to the transcriptomics encoder. The two modalities are then aligned in a shared representation space using a contrastive learning objective. GigaTIME (tumor immune microenvironment) is a multimodal foundation model that translates H&E images into multiplex immunofluorescence (mIF) spatial proteomics, enabling cost-effective profiling of the TIME in clinical settings.^162^ GigaTIME is pre-trained on 40 million paired cells and H&E patches across 21 protein markers and employs a cross-modal encoder-decoder based on the NestedUNet (CNN-based) architecture.

### Practical guidelines for method selection

The proliferation of computational tools for multimodal spatial omics integration creates a practical challenge for users seeking to identify the most appropriate method for their specific experimental design. To address this, we propose a set of practical guidelines for method selection based on key data characteristics and computational constraints.

When the primary goal is cell-type deconvolution and a well-annotated scRNA-seq reference is available, probabilistic methods such as Cell2location and Stereoscope offer principled uncertainty quantification and are well suited for Visium-resolution data.^96^^,^^97^ However, as deconvolution performance relies on the quality of the reference data, a matched scRNA-seq dataset is ideal. For spatial domain identification tasks involving the integration of multiple spatial modalities (e.g., ST with spatial proteomics or epigenomics), graph-based methods such as SpatialGlue and MISO are recommended, as they can naturally encode spatial neighborhood structure and can handle heterogeneous feature types. When computational resources are limited, datasets are small, or interpretability is essential, matrix factorization approaches (e.g., CellPie and LIGER) provide not only lightweight, scalable alternatives that are also interpretable. For cross-section alignment tasks where serial sections must be co-registered, optimal transport methods such as PASTE and Moscot offer geometrically principled alignment. In exploratory settings, where the goal is to predict spatial omics from H&E images, deep learning architectures such as CNNs, GCNs, or ViTs are most appropriate, although they typically require larger training datasets. However, many of these methods are pre-trained on specific spatial omics technologies and tissue types, which can limit their applicability to new tissues and experimental platforms. To address this, researchers often rely on transfer learning, domain adaptation, or fine-tuning strategies that leverage pre-trained models. Finally, when data suffer from severe dropout noise, likelihood-based frameworks that explicitly model zero-inflation and overdispersion, such as those employing NB or zero-inflated NB (ZINB) observation models, are particularly appropriate, as they distinguish technical dropouts from genuine biological zeros and thereby avoid inflating downstream integration artifacts.^163^ Alternatively, preprocessing strategies, such as restricting analysis to highly expressed or highly variable features, can further improve robustness in these settings (Table 2).

**Table 2:** Decision matrix for computational method selection in multimodal spatial omics integration

Category	Scenario	Recommended approach	Advantages	Limitations
Core analytical tasks	cell-type deconvolution	probabilistic modeling, e.g., *Cell2location, Stereoscope*	uncertainty quantification; suited for Visium data	requires a high-quality scRNA-seq reference; ideally matched data
	spatial domain identification	graph neural networks, e.g., *SpatialGlue, MISO*	integrates modalities, captures spatial neighborhood structure, handles heterogeneity	higher computational cost, depends on data quality, spatial oversmoothing
	cross-section alignment	optimal transport based, e.g., *PASTE, Moscot*	preserves spatial geometry	computationally intensive
	prediction from H&E	deep learning, e.g., *CNNs, GCNs, ViTs*	predicting expensive omics modalities from routine pathology; flexible modeling	requires large datasets, limited generalization
Practical considerations	resource-limited/interpretable	matrix factorization, e.g., *CellPie, LIGER*	lightweight, scalable, interpretable factors	may miss complex non-linear patterns
	generalisation	transfer learning, domain adaptation, fine-tuning	extends pre-trained models to new datasets	computational cost depends on the domain shift and dataset
	dropout handling	NB/ZINB models, preprocessing strategies, e.g., *Cell2location, Stereoscope, DestVI Hist2ST*	models dropout implicitly via overdispersion; improved robustness	model assumptions required, added complexity

Recommended algorithmic families and representative tools based on the primary computational task, data characteristics, and practical constraints.

## Challenges in spatial multimodal acquisition and integration

Despite remarkable progress in spatial multi-omics technologies and computational methods, several fundamental challenges must be addressed before these approaches can achieve their full potential in basic research and clinical translation.

### Technical limitations and standardization

Simultaneous measurement of multiple molecular layers from the same tissue section remains technically challenging due to assay compatibility and tissue preservation. Sequential processing steps can compromise data quality; for example, immunostaining protocols may degrade RNA integrity for subsequent transcriptomic profiling, while fixation methods optimized for proteomics may not preserve metabolite distributions.^85^^,^^86^ Co-profiling technologies such as DBiT-seq, MISAR-seq, and spatial ATAC-RNA-seq require careful optimization and are not universally compatible across tissue types or preservation methods. The technical complexity of preserving RNA, proteins, chromatin accessibility, and metabolites simultaneously in a single section presents a fundamental biochemical challenge that requires continued protocol development.

A second major challenge arises from differences in spatial resolution across modalities. Different spatial modalities operate at fundamentally different resolutions: array-based transcriptomics comes at a multicellular resolution, whereas MSI achieves near-single-cell resolution,^44^^,^^49^ and imaging-based transcriptomics platforms (MERFISH and Xenium) provide subcellular resolution. This resolution mismatch complicates integration, as high-resolution modalities capture cellular and subcellular heterogeneity obscured in lower-resolution measurements. For instance, integrating 2 μm resolution IMC data with 55 μm Visium spots requires aggregating the former and deconvolving the latter (Box 1), both of which introduce uncertainties. Emerging approaches—including tissue expansion microscopy,^21^ high-density microfluidic barcoding,^10^^,^^11^ and high-resolution ST platforms—are narrowing this gap, though complete resolution harmonization across all modalities remains elusive.

Building on these cross-modal integration challenges, additional difficulties arise at the level of individual spatial modalities. Spatial epigenomics technologies generate inherently sparse data—chromatin accessibility and histone modification measurements exhibit severe zero-inflation even at single-cell resolution. This sparsity is further exacerbated in tissue sections, requiring spatial aggregation across neighborhoods to achieve statistical power while potentially masking fine-grained regulatory heterogeneity.^164^ Spatial metabolomics via MSI faces distinct challenges in metabolite identification due to ion suppression effects, matrix interferences, limited spectral reference databases, and the immense chemical diversity of metabolites.^69^ Unlike genes or proteins, which have defined sequences, metabolites lack universal barcodes, making confident annotation difficult. This is a particular challenge for lipids, due to their large numbers (as the dominant metabolite class) and structural diversity. Moreover, integrating metabolomics with transcriptomics or proteomics is complicated by indirect correspondence—metabolite levels reflect enzyme (protein) concentrations and activity, substrate availability, transport, and turnover rather than simply gene expression. Current strategies to address this gap include geometry-driven co-registration (e.g., MIIT; see section “OT and geometric alignment”), pathway-informed approaches that exploit enzyme-metabolite relationships to link gene expression with metabolic output, and latent-space methods such as MISO that learn shared representations via spatial proximity without requiring direct molecular anchors.^142^ Nonetheless, moving beyond correlation-based frameworks toward pathway-constrained models that encode biochemical knowledge remains a critical need.

Multimodal spatial profiling is also constrained by cost and scalability. Complete tissue characterization combining transcriptomics, proteomics, epigenomics, and metabolomics often exceeds £5,000–£10,000 per specimen when accounting for reagents, instrument time, and computational analysis. Specialized instrumentation requirements limit accessibility to well-resourced core facilities and institutions. These capital and operational barriers constrain large-cohort epidemiological studies, longitudinal disease monitoring, and clinical implementation where cost-per-sample budgets are stringent.

In addition, the field currently lacks consensus protocols for sample preparation, quality control metrics, and data reporting standards. Laboratory-specific workflows hinder cross-study comparisons and reproducibility—one group’s ST may use fresh-frozen tissue with Visium, while another uses FFPE with GeoMx, yielding fundamentally different data characteristics.^165^ Batch effects arising from differences in tissue handling, fixation duration, sectioning thickness, staining protocols, and reagent lots are pervasive. Similarly, H&E imaging lacks standardized protocols for scanning resolution, color normalization, and image preprocessing. Variations in scanner hardware, objective magnification, compression algorithms, and staining intensity across institutions introduce batch effects that confound downstream imaging-based predictions and cross-study integration.

Community-driven initiatives to establish best practices, reference materials (e.g., spike-in controls and tissue standards), and metadata annotation guidelines—analogous to efforts by the Human Cell Atlas^166^ in single-cell genomics—are urgently needed to enable meta-analyses and accelerate method development through rigorous benchmarking. Encouragingly, early standardization efforts are emerging in specific subfields. For mIF and immunohistochemistry, the Society for Immunotherapy of Cancer recently published the STORMI (Standards for Reporting of Multiplex Immunohistochemistry/Immunofluorescence Assays) consensus checklist, which defines the requirements for reporting analytical validation, image acquisition and registration, cell clustering, and spatial analysis strategies.^167^ These reporting standards complement multi-institutional reproducibility studies such as the MITRE (multi-institutional TSA-amplified multiplexed immunofluorescence reproducibility evaluation) study, which demonstrates that standardized mIF protocols can achieve high inter-site concordance across six cancer centers, supporting clinical deployment of spatially resolved immune profiling.^168^ Similarly, the AstroPath platform—which adapts astronomical sky-mapping algorithms to multiplex pathology—has provided an end-to-end, quality-controlled workflow for quantitative spatial analysis of the TIME and has been validated in multi-site clinical studies of anti-PD-1 immunotherapy response.^169^^,^^170^ These initiatives demonstrate the feasibility of standardization and provide a template that the broader spatial multi-omics community should follow.

### Computational complexity and data management

As technology resolution and multimodality increase, spatial omics datasets also impose substantial computational complexity and data management challenges. Individual spatial multi-omics experiments generate terabyte-scale datasets. For example, a single tissue section profiled by Visium paired with high-resolution H&E imaging, IMC proteomics, and MALDI metabolomics can easily exceed ∼1–5 *TB* of raw and processed data. Longitudinal studies, cohort analyses, or 3D tissue reconstructions from serial sections amplify these demands. Data storage, transfer, and long-term archiving present non-trivial infrastructure challenges, particularly for academic laboratories without dedicated bioinformatics cores or cloud computing budgets. Standardized, compressed file formats (analogous to BAM for sequencing), and public data repositories with cloud-accessible storage are needed.

Beyond infrastructure, the scale of spatial multi-omics data demands computationally scalable methods. Many state-of-the-art approaches—including deep learning architectures and Bayesian inference frameworks—require extensive training data and computational resources that may be prohibitive for smaller laboratories or exploratory studies. Developing lightweight, sample-efficient algorithms alongside data-hungry foundation models remains a critical priority.

However, computational scalability and data management are only one aspect; robust biological inference and interpretation are further complicated by modality-specific noise. This includes sequencing dropouts and amplification biases in transcriptomics, antibody cross-reactivity and autofluorescence in imaging, and matrix effects and ionization variability in MSI. When integrating noisy measurements across modalities, there is a risk of either amplifying technical artifacts (if modalities share systematic biases) or diluting biological signals (if noise is uncorrelated). Distinguishing true cross-modal relationships from spurious correlations requires robust statistical frameworks that explicitly model modality-specific noise structures.^163^ For example, apparent mRNA-protein discordance may reflect technical noise rather than post-transcriptional regulation, necessitating uncertainty quantification in integration outputs.

In addition, spatial multimodal omics analysis is hindered by fragmented and heterogeneous computational pipelines. Current workflows typically involve stitching together disparate software tools: image preprocessing (ImageJ^171^ and QuPath^172^), ST quantification (Space Ranger^173^ and STARmap tools^174^), H&E feature extraction (HistoQC^175^), MSI processing (Cardinal^176^ and SCiLS), quality control (custom scripts), and integration methods. This heterogeneity creates steep learning curves, introduces opportunities for error at each step, and impedes accessibility for scientists without computational expertise. Development of unified, end-to-end pipelines with standardized inputs/outputs—such as Squidpy^177^ and SpatialData,^178^ an open and universal framework for processing spatial omics data—would democratize spatial multi-omics analysis. Cloud-based platforms offering interactive analysis (e.g., Galaxy for spatial data) could further improve accessibility.

### Integration validation and benchmarking

As outlined in Box 1, spatial multi-omics analysis relies on multiple interconnected computational tasks: cell-type deconvolution, spatial domain identification, cross-modal co-registration, histology-based molecular prediction, and gene imputation. However, validating the accuracy of these methods remains a fundamental challenge.

The proliferation of integration algorithms creates a paradox of choice.^92^ Methods vary in their assumptions, computational requirements, and suitability for different experimental designs. Clear guidelines for method selection based on data characteristics, sample size, modality types, resolution mismatches, and batch structure are still lacking. Practitioners often resort to trial-and-error or follow the most-cited method, which may not be optimal for their specific use case.

A fundamental limitation arises from the absence of ground-truth labels for evaluating spatial multimodal integration algorithms. For example, when mRNA and protein measurements disagree on cell-type assignment at a spatial location, determining which modality better represents the ground-truth—or whether both capture complementary but valid information (e.g., mRNA in the cell body and protein in projections)—is non-trivial. This challenge is particularly acute in diagonal integration, where modalities share no common anchor (e.g., transcriptomics from donor A and proteomics from donor B).^5^ Without known truth, validation relies on indirect proxies: consistency with orthogonal measurements (immunofluorescence confirming predicted protein), alignment with published cell-type markers, preservation of expected spatial autocorrelation, or biological plausibility of inferred relationships. These heuristics are valuable but cannot definitively prove correctness.

Although ground-truth annotations are not always available, the spatial omics community has converged on a set of quantitative metrics that serve as proxies for assessing integration quality. For cell-type deconvolution, metrics such as the root mean square error (RMSE), mean absolute error (MAE), and Pearson or Spearman correlation between predicted and reference cell-type proportions are commonly used when synthetic or semi-synthetic benchmarks are available.^179^ Distributional metrics, including the Jensen-Shannon divergence (JSD) and KL divergence, are also employed to quantify the agreement between predicted and observed spatial cell-type distributions, while the structural similarity index (SSIM), originally developed for image quality assessment, quantifies the similarity between predicted and reference mappings. For spatial domain identification and clustering, evaluation metrics such as the adjusted rand index (ARI), mutual information (MI), normalized mutual information (NMI), and the Silhouette score are widely used. Metrics such as ARI and NMI require ground-truth annotations, which often originate from expert histopathological labeling or known anatomical structures, whereas the Silhouette score evaluates cluster compactness and separation in the learned embedding space. Spatial coherence metrics are additionally used to assess whether inferred domains form biologically plausible spatial structures, including measures of neighborhood consistency and spatial autocorrelation statistics such as Moran’s I or Geary’s C applied to domain labels. For co-registration and spatial alignment tasks, alignment accuracy is typically quantified using landmark correspondence errors, such as the target registration error (TRE), which measures the Euclidean distance between manually annotated landmarks in the fixed and warped images. When ground-truth alignment or synthetic datasets are available, reconstruction metrics such as mean square error (MSE) between the inferred and reference data are also reported. Additional metrics include the label transfer adjusted rand index (LTARI), which evaluates whether labels such as cell types can be correctly transferred between slices following alignment, and the Dice coefficient, which measures the overlap between co-registered tissue masks. For spatial omics prediction tasks from histology images, Pearson correlation per gene or modality is commonly used alongside error-based metrics, including MAE, MSE, and RMSE, as well as the fraction of genes with statistically significant prediction performance. For classification-based tasks, most studies report the area under the receiver operating characteristic curve (AUC-ROC), together with accuracy, precision, recall, and F1-score to quantify predictive performance. Finally, for gene expression imputation, cosine similarity and Pearson or Spearman correlation between imputed and held-out expression profiles are frequently used as quantitative benchmarks. Despite this growing repertoire of evaluation metrics, the field still lacks consensus on a standardized benchmarking framework, and many studies report only a subset of these measures, which hinders systematic cross-method comparison across spatial omics integration methods.

Rigorous benchmarking studies are essential to evaluate integration accuracy and identify failure modes. Ideal benchmarks would include: (1) simulated datasets with known ground-truth (e.g., in silico mixtures of single-cell references with defined cell-type compositions and spatial patterns), (2) spike-in controls where synthetic barcodes or proteins are added at known locations, (3) orthogonal validation cohorts profiled by entirely independent technologies, and (4) perturbation experiments where biological ground-truth is established (e.g., genetic knockout verified by genomic sequencing).^179^ Community challenges—like DREAM competitions^180^ or CAFA^181^ in systems biology—could crowdsource method development and establish performance baselines across diverse tissue types and biological questions.

### Biological interpretation and multi-scale integration

Interpreting multi-omics patterns requires integrating knowledge across molecular layers with distinct regulatory relationships. Chromatin accessibility enables transcription factor binding; mRNA abundance influences but does not determine protein levels due to translational regulation and protein stability; and protein or enzyme concentrations and activity, together with substrate availability, determine metabolite transformations.^182^ Deviations from expected layer-to-layer correlations may reflect biologically meaningful regulation (e.g., miRNA-mediated post-transcriptional silencing causing mRNA-protein discordance) or technical artifacts (e.g., antibody cross-reactivity inflating protein measurements). Adjudicating these scenarios demands mechanistic pathway knowledge, time series data to infer directionality, and perturbation experiments to establish causality—capabilities often unavailable in cross-sectional spatial studies.

Tissues exhibit nested hierarchical structures: molecules within subcellular compartments, compartments within cells, cells within microenvironmental niches, and niches within anatomical regions. Observed spatial patterns may arise from multiple non-mutually exclusive mechanisms: cell-intrinsic transcriptional programs (e.g., zonated hepatocyte metabolism), local cell-cell signaling (paracrine gradients), or tissue-scale physical forces (oxygen/nutrient gradients). Disentangling these influences requires multi-scale models that partition variance across spatial length scales and experimental perturbations that isolate specific mechanisms.^111^ Recent computational frameworks have begun to model multi-scale spatial organization. For example, SPATIA explicitly models multi-scale biological data via a hierarchical architecture that integrates cellular, local tissue, and global tissue-level features, while Nicheformer maps dissociated single-cell datasets onto spatial contexts across biological scales. MNiST^183^ captures multi-scale spatial structure through frequency patterns, spatial topological features, and mappings between expression distributions. MNiST is based on Gmamba, a dual-branch encoder to capture both local and long-range spatial dependencies within tissues, while frequency-domain representations using Chebyshev polynomial expansion enable multi-order analysis that captures structural and boundary information across scales. Similarly, SCALE^184^ uses a GNN-based encoder-decoder architecture to identify spatial domains across multiple resolutions. Complementary to deep learning approaches, CRAWDAD^185^ statistically quantifies spatial relationships between cell types across spatial length scales by comparing observed neighborhood cell-type proportions with spatially shuffled backgrounds. Together, these approaches highlight the growing recognition that spatial biological organization is inherently multi-scale, suggesting that future computational frameworks should explicitly integrate information across scales within multiple spatial omics layers.

Effective communication of spatial multi-omics results to diverse audiences—computational biologists, experimental researchers, clinicians, and the public—requires intuitive visualization strategies. Challenges include (1) dimensionality: conveying information across genes, proteins, metabolites, and spatial coordinates simultaneously; (2) scale: enabling exploration from tissue-level maps down to single-cell detail; and (3) interactivity: allowing users to query specific genes/proteins/metabolites, toggle modalities, and overlay clinical metadata.^186^ Interactive tissue atlases^187^^,^^188^ with searchable gene/protein/metabolite databases, linked spatial maps (e.g., Allen Brain Atlas style interfaces)^189^ and 3D tissue reconstructions^174^ from serial sections represent the state of the art. However, many published datasets lack accessible visualization portals, limiting their utility for hypothesis generation by the broader community.

#### Future outlook

Realizing the full translational potential of spatial multimodal omics requires moving beyond descriptive mapping to predictive modeling. Key aspirational goals include in silico perturbation, which involves simulating how genetic knockouts, drug treatments, or microenvironmental changes would propagate across molecular layers in spatial context; biomarker discovery, which focuses on identifying minimal gene, protein, or metabolite signatures that predict clinical outcomes from spatial architecture, and digital twins, which aim to construct mechanistic models of tissues that recapitulate observed spatial multimodal omics data and enable counterfactual queries.^190^ These applications demand integration of spatial multi-omics with dynamic models (ordinary/partial differential equations and agent-based simulations) and causal inference frameworks—methodological frontiers that remain largely unexplored.

Despite these challenges, the field is poised for transformative advances. Lessons from single-cell genomics—where analogous challenges in scalability, batch correction, and multimodal integration were systematically overcome through community collaboration, open data sharing, and sustained investment—provide an encouraging roadmap.

Key future directions include the establishment of standardized protocols and reference datasets, incorporating consensus sample preparation workflows, spike-in quality control standards, and benchmark datasets with both simulated and experimentally validated ground-truth annotations to enable rigorous method comparison. In parallel, the expansion of open data repositories, such as spatial extensions of the Human Cell Atlas^166^ and the Human Tumor Atlas Network,^191^ will be critical, particularly when accompanied by rich, high-quality metadata annotation (including tissue provenance, processing protocols, and clinical covariates) and scalable, cloud-accessible infrastructure to support meta-analyses and machine learning applications. Alongside these data-centric efforts, user-friendly analysis platforms are needed to lower barriers to adoption, integrating existing ecosystems such as Seurat, Scanpy, Giotto, and Squidpy with graphical interfaces, automated quality control, and standardized best-practice pipelines.

From a translational perspective, clinical adoption will depend on reducing per-sample costs through automation, miniaturization, and economies of scale, while also streamlining workflows to meet clinical time constraints, such as rapid diagnostic turnaround and establishing regulatory frameworks for spatial omics-based diagnostics. Finally, sustained progress will require stronger interdisciplinary collaboration between technology developers, computational method developers, biological researchers, and clinicians through consortia, workshops, and collaborative funding mechanisms, ensuring that methodological advances are driven by real biological and clinical needs rather than computational benchmarks alone.

As experimental resolution improves, costs decline, and computational methods mature, spatial multimodal omics will transition from a specialized research tool to a routine approach for dissecting tissue biology. The convergence of nanoscale imaging, single-molecule sequencing, machine learning, and mechanistic modeling positions the field to deliver on its promise of revolutionizing our understanding of tissue organization in development, homeostasis, and disease—ultimately enabling precision medicine grounded in the spatial architecture of human tissues.

## Acknowledgments

S.G. and Y.H.U. are supported by the University of Manchester Research Institute (UMRI) Pump Priming Award. M.R. is supported by the AI-hub in Generative Models (EPSRC ref. EP/Y028805/). E.B.I. is funded by the Manchester ELLIS Unit. A.N. acknowledges 10.13039/501100000268BBSRC (BB/W006022/1). H.Z. acknowledges 10.13039/501100000289CRUK (PRCBTP-Nov24/100012).

## Author contributions

Conceptualization, E.B.I., Y.H.U., and S.G.; writing – original draft, E.B.I., Y.H.U., S.G., M.R., H.L., and W.R.; writing – review and editing, E.B.I., Y.H.U., H.L., W.R., H.Z., A.N., M.R., and S.G.; and funding acquisition, S.G. and M.R.

## Declaration of interests

The authors declare no competing interests.
